# Putative prophages related to lytic tailless marine dsDNA phage PM2 are widespread in the genomes of aquatic bacteria

**DOI:** 10.1186/1471-2164-8-236

**Published:** 2007-07-16

**Authors:** Mart Krupovič, Dennis H Bamford

**Affiliations:** 1 Department of Biological and Environmental Sciences and Institute of Biotechnology, Biocenter 2, P.O. Box 56 (Viikinkaari 5), 00014 University of Helsinki, Finland

## Abstract

**Background:**

The origin and evolution of viruses is currently a heavily discussed issue. One element in this discussion is the innate viral "self" concept, which suggests that viral structures and functions can be divided into two categories. The first category consists of genetic determinants that are inherited from a viral ancestor and encode the viral "self". The second group consists of another set of structures and functions, the "nonself", which is interchangeable between different viruses and can be obtained via lateral gene transfer. Comparing the structures and sequences of the "self" elements, we have proposed that viruses can be grouped into lineages regardless of which domain of life (bacteria, archaea, eukarya) they infect. It has also been suggested that viruses are ancient and possibly predate modern cells.

**Results:**

Here we identified thirteen putative prophages (viral genomes integrated into bacterial chromosome) closely related to the virulent icosahedral tailless lipid-containing bacteriophage PM2. Using the comparative genomics approach, we present evidence to support the viral "self" hypothesis and divide genes of the bacteriophage PM2 and related prophages into "self" and "nonself" categories.

**Conclusion:**

We show here that the previously proposed most conserved viral "self" determinants, the major coat protein and the packaging ATPase, were the only proteins that could be recognized in all detected corticoviral elements. We also argue here that the genes needed for viral genome replication, as well as for host cell lysis, belong to the "nonself" category of genes.

Furthermore, we suggest that abundance of PM2-like viruses in the aquatic environment as well as their importance in the ecology of aquatic microorganisms might have been underestimated.

## Background

Recently observed structural relationships between the architectures of viruses infecting hosts from the three domains of life [1-4] poured fuel onto the discussion of the origin and evolution of viruses [5-11]. It is now evident that viruses are ancient and that very diverse virus families may have arisen from a relatively small number of primordial viral ancestors. We have suggested previously that a limited number of features represent the essence of a virus and that viruses sharing such characteristics can be joined into lineages. Those features are responsible for the formation of a functional virion and are referred here as the viral "self" (discussed in [5,6,8,12,13]). On the other hand, components encoded in viral genome and needed for interaction with the host are assumed to be nonessential for the virion formation and thus represent the "nonself" category, although they are indispensable for viral genome replication and the interaction of the virus with its host.

Ascribing viral properties to "self" or "nonself" categories for highly divergent viruses, which nonetheless, belong to the same viral lineage [12], is based on structural and functional relationships. However, these relationships often cannot be revealed using sequence analysis due to the lack of detectable similarity. Components of the viral "self" in more closely related viruses are expected to be conserved also at the sequence level. The first direct support for the presence of the "self" elements was obtained when genomes of six tectiviruses were compared [13]. The analysed viruses were highly similar (overall identities between 91.9% and 99.8%), but there were clear differences in the number of altered nucleotides between the genes. Genes for structural elements and those for genome packaging were the most conserved, whereas the virus entry and release-related genes were the least conserved ones [13].

Bacteriophage PM2, the only known member of the *Corticoviridae *virus family [14], is a tailless marine bacteriophage that was isolated from the coastal seawater in Chile [15]. There are so far only two closely related *Pseudoalteromonas *species, *Pseudoalteromonas espejiana *BAL-31 and *Pseudoalteromonas *sp. ER72M2, which PM2 was shown to infect [16,17]. The PM2 virion consists of an icosahedrally organized proteinaceous capsid surrounding a protein-rich lipid membrane, which encloses a highly supercoiled circular double-stranded DNA (dsDNA) genome [18-20]. Replication of the viral genome takes place near the cytoplasmic membrane via a rolling-circle mechanism initiated by the phage-encoded replication initiation protein P12 [19,21]. The 10 079 bp-long PM2 genome is organised into three operons – two early and one late [22]. The two early operons are transcribed in opposite directions. The leftwards-transcribing early operon encodes transcriptional repressors (P15 and P16, genes *XV *and *XVI*, respectively), while the rightwards-transcribing early operon codes for protein(s) required for genome replication, transcription activators of the late operon (proteins P13 and P14), as well as one structural protein, the packaging ATPase P9. The rest of the structural protein-coding genes (genes *I*-*VIII *and *X*) as well as genes (*XVII*, *XVIII*) encoding proteins involved in the cell lysis are located in the late rightwards-transcribing operon and are tightly packed. PM2 is a virulent phage and has not been shown to lysogenize its host cells.

In this study we identified thirteen PM2-like elements residing in the genomes of eleven aquatic proteobacteria. Comparative analysis of gene composition between the identified putative prophages and bacteriophage PM2 let us to test and support the viral "self" hypothesis. Furthermore, our results show that PM2-like elements are abundant in the genomes of aquatic bacteria and therefore the role of tailless bacteriophages on aquatic microorganisms might be underestimated.

## Results and discussion

Putative corticoviral prophages were identified by homology-based searches against the nonredundant protein database at NCBI. For our search, we selected gene encoding the canonical viral "self" element, the major coat protein (MCP) P2 [20]. In all of these cases, genes for the packaging ATPase P9 [20,23,24] homologues were observed in the proximity of the P2-encoding genes. It should be noted that this approach enables detection of only close phage relatives that have not yet diverged to a point where homologous sequences are no longer recognizable [6]. Thirteen homologues of P2-encoding genes were identified in eleven bacterial genomes. PM2 genome is 10 079 bp-long, therefore an approximately 15 kb-long region surrounding the P2/P9-encoding pair of genes was further examined for the presence of other putative PM2-related genes.

When protein sequences homologous to the MCP of bacteriophage PM2 were assayed for structure prediction, they all matched, as the first hit, to the MCP of the Paramecium bursaria chlorella virus 1 (PBCV-1) [4], although the scores were low (below the significance value, E > 1). Interestingly, validation for this observation was obtained from the ongoing structural characterization of the MCP P2 of phage PM2 [25]. Our preliminary results indicate that P2 has the same topology as the other members of the PRD1-adeno viral lineage [Abrescia et al., to be published], which among others also includes PBCV-1 [8].

### Analysis of the corticoviral elements

**Figure 1:**
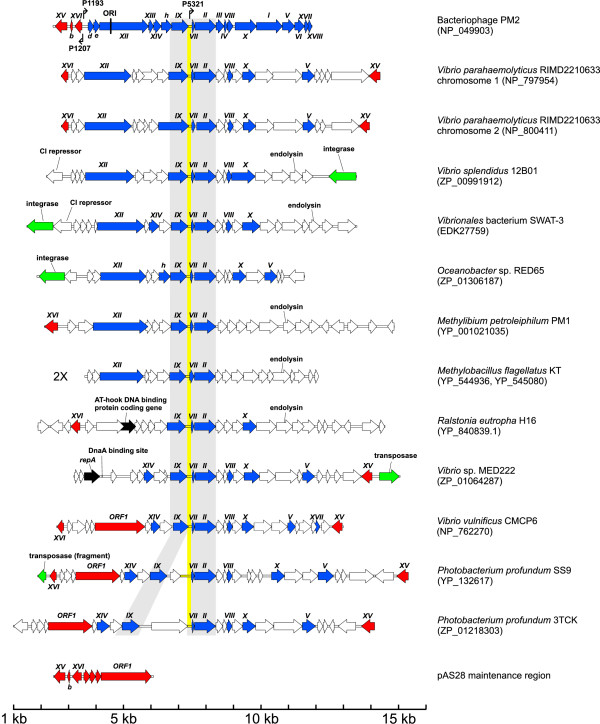
Genetic organization of the putative corticoviral prophages, bacteriophage PM2 [19], and the maintenance region of the pAS28 plasmid [26]. Circular PM2 genome map is linearized at a unique EcoRII site [19]. Genes encoding structural and nonstructural proteins (Roman numerals) and ORFs (lower case letters) are visualized as arrows indicating the direction of transcription. The origin of replication (ORI) and the known promoters (kinked arrows) are indicated. All the elements are aligned to the major coat protein-coding gene *II *of phage PM2, except for the pAS28 maintenance region, which is aligned to the PM2 gene *XVI*. Genes in the corticoviral elements sharing sequence similarity with those of PM2 are named according to the previously established phage PM2 nomenclature [19] and are coloured blue. Genes in the PM2 genome, as well as in corticoviral elements related to those of the pAS28 plasmid, are coloured red. The viral "self" core, containing genes *IX*, *VII*, and *II *is shaded grey. The late promoter region of phage PM2 and the noncoding region matching its position in all putative prophages is coloured yellow. Recombinase-coding genes and genes encoding replication-associated proteins (other than *XII*- and *ORF1*-type) are shown in green and black, respectively. Putative CI repressor- and endolysin-coding genes, as well as the position of the DnaA protein-binding site, are indicated. As a reference point, accession numbers of putative coat proteins of the identified corticoviral prophages are indicated in parenthesis under the corresponding bacterial species name. While exact nucleotide positions of the depicted corticoviral elements can be found in the supplemental material table (see Additional file 1).

Four functionally distinct groups of genes constitute the PM2 genome. These are genes encoding proteins responsible for genome replication (replication initiation protein P12), transcription regulation (transcription factors P13–P16), structural components of the virion (proteins P1–P10), and proteins involved in cell lysis (P17, P18). The core viral "self" determinants, genes *II *(protein P2) and *IX *(protein P9), are separated by the late promoter region and a short gene *VII *coding for structural protein P7. An open reading frame (ORF) corresponding to gene *VII *as well as an intergenic region matching the PM2 late promoter (coloured yellow in Fig. 1) were also recognized in all thirteen putative prophages. The region coding for gene *II*/*IX *homologs was always found as a block, exactly as in the PM2 genome, except in *Photobacterium profundum *species SS9 and 3TCK, where the putative prophages contained an additional ORF (Fig 1). Genes coding for structural proteins P5, P8, and P10 were found in 54%, 62%, and 77% of putative corticoviral elements, respectively (Fig. 1). Notably, *Vibrio parahaemolyticus *RIMD2210633 contained two copies of the PM2-like element, one in each of its two chromosomes, while *Methylobacillus flagellatus *KT had two absolutely identical (at the nucleotide level) elements in its single chromosome.

As mentioned above, the PM2 genome is organised into three operons – two early and one late [22]. The maintenance region of *Pseudoalteromonas *plasmid pAS28 [26] was shown to share similar genomic organisation with the two early transcriptional units of PM2 [22]. Significant sequence similarity was found only between the leftwards-transcribing operons and the promoter regions (see Fig. 1). Replication protein coding genes of the pAS28 plasmid (*ORF 1*) and phage PM2 (gene *XII*) did not share any sequence similarity, although they were located at the equivalent positions (see Fig. 1). In light of those observations, it was proposed that the entire leftwards-transcribing early operon together with the promoter region was obtained from the *Pseudoalteromonas *plasmid pAS28 via horizontal gene transfer [22]. Both genes, *XV *and *XVI*, were detected in putative prophages of *Vibrio parahaemolyticus*, *Vibrio vulnificus *CMCP6, and *Photobacterium profundum *SS9, where they were flanking the rest of the viral genes. The remaining corticoviral elements (four out of nine) contained either gene *XV *or *XVI*. Interestingly, it seems that in all cases the presumably circular genomes of the corticoviral elements were linearized between the gene *XV *and *XVI*. Gene *XV *was shown to be dispensable in laboratory conditions [27], however, its presence in the genomes of the putative prophages suggests that it might play a beneficial role for the phage in the natural environment.

Surprisingly, gene *XII*-type replication initiation protein genes were identified only in six of the eleven bacterial species (PM2-type replication system), and *ORF 1*-type (as in plasmid pAS28) replication protein genes were identified in three of the corticoviral elements (pAS28-type replication system). Putative prophage of *Vibrio *sp. MED222 encoded an ORF, the product of which shared sequence similarity (23% identity, 59% similarity) with a unique replication protein RepA (accession no. YP_025331) from *Pseudomonas alcaligenes *plasmid pRA2 (pRA2-type replication system) [28]. Furthermore, an identical possible DnaA protein binding site, 5'-TTATCCACA-3', essential for pRA2 plasmid replication, was also found in the putative prophage sequence at the equivalent position, downstream of the *repA *gene. No putative replication protein-coding genes were identified in the *Ralstonia eutropha *H16 corticoviral element. However, we identified an ORF coding for a potential DNA binding protein containing the so-called AT-hook motif [29]. The AT-hook motif has been shown to be required for the replication and partitioning of Epstein-Barr virus *oriP *plasmids [30], as well as for the origin recognition complex binding to the replication origin in fission yeast [31]. Therefore, it is possible that the AT-hook protein might be involved in the replication of the corticoviral element in *Ralstonia eutropha *H16, thus comprising the forth replication system (AT-hook-type) found in PM2-like elements. Furthermore, the AT-hook motif-containing protein in *Ralstonia eutropha *H16 shares sequence similarity with AT-hook motif proteins from *Ralstonia pickettii *filamentous bacteriophage p12j (accession no. AAQ90254; 35% identity; 54% similarity) and *Ralstonia solanacearum *plasmid pJTPS1 (accession no. BAA32222; 43% identity, 67% similarity), suggesting possible genetic exchanges between those three *Ralstonia *elements. *Vibrio vulnificus *CMCP6 has two chromosomes. The putative corticoviral prophage was found on chromosome 2 only. This prophage has the *ORF 1*-type replication protein-coding gene. On the other hand, chromosome 1 contains a gene, encoding a putative homolog of the replication initiation protein P12 (NP_761302, E = 1e-65), but no other PM2-related genes. It is possible that a genetic exchange event between the two chromosomes of *Vibrio vulnificus *CMCP6 gave rise to viruses with the gene *XII*-type replication initiation protein-coding genes.

We have previously noticed that in the same viral lineage (viruses having a similar mechanism to assemble the virion) replication systems do not need to be related, i.e. the "self" and the replication systems are uncoupled. The final replication product just has to be specifically recognized for genome packaging [12]. This observation is supported here by the finding that putative PM2-like prophages encode several types of replication systems. Consequently, genes for genome replication cannot be considered as being part of the viral "self" category, although such genes are among the most conserved ones in viral genomes. This is also true for the tailed dsDNA bacteriophages (order *Caudovirales*). It has been observed that members of the *Podoviridae *family use two different strategies to replicate their genomes [32]. T7-like phages replicate their genomes by a primase/DNA polymerase mechanism, while φ29-like podoviruses use a covalently linked 5' terminal protein primer [33]. Both, T7 and φ29, have the canonical HK97 MCP fold [34-36]. Interestingly, icosahedral tailless lipid-containing phage PRD1, a type member of the *Tectiviridae *family, also uses the latter strategy to replicate its linear dsDNA genome [37], but has a completely different MCP topology [1].

In order to escape from the infected cell, bacteriophages have to overcome the cell envelope barrier. All dsDNA bacteriophages characterized so far use the holin-endolysin system to accomplish this step [38]. Holins are small phage-encoded membrane proteins that, in a precisely scheduled manner, form nonspecific lesions in the plasma membrane of the infected cell. Endolysins are phage-encoded peptidoglycan-degrading enzymes that may possess endo-β-N-acetylglucosaminidase, N-acetylmuramidase, N-acetylmuramoyl-L-alanine amidase, and/or endopeptidase activities. The endolysins are translocated to the periplasmic space or are activated with the aid of holin proteins. However, no putative endolysins were recognized in the PM2 proteome. Instead, we identified genes (*XVII *and *XVIII*) that encode novel lysis-associated proteins P17 and P18 [27,39]. We also discovered that P18 homologues are encoded in the genomes of several enteric (PaP3, Felix 01, and RB49) and marine (P-SSM4, S-PM2, and P-SSM2) tailed bacteriophages [39]. This illustrates that lysis genes may be exchanged between bacteriophages belonging to different lineages. Only the putative prophage of *Vibrio vulnificus *CMCP6 contained a homolog of PM2 gene *XVII *(32% identity, 82% similarity at the amino acid level), while no homologues of gene *XVIII *could be identified. Interestingly, putative endolysin genes, similar to those of other dsDNA bacteriophages, were found in corticoviral elements of *Vibrio splendidus *12B01 and *Vibrionales *bacterium SWAT-3 (hits to putative endolysin protein [NP_891673] of phage RB49; E = 4e-31 and E = 1e-30, respectively), *Methylobacillus flagellatus *KT (lysozyme [NP_690649] of phage B103; E = 4e-27), *Methylibium petroleiphilum *PM1 (putative lysozyme [NP_958065] of phage PsP3; E = 1e-25), and *Ralstonia eutropha *H16 (lysin protein [NP_599082] of phage SfV; E = 1e-28) (Fig. 1). Lysis genes have not been shown to participate in virion assembly, e.g. interfere with the "self" function. As shown here, the same family of viruses may have a very different set of genes responsible for progeny release. Consequently, similarly to the replication systems, the lysis-related genes belong to the viral "nonself" category.

In order to establish a stable relationship with the host organism, temperate phages replicate in synchrony with the bacterial chromosome. One mechanism to do so is to integrate the phage genome as part of the host chromosome. This event is catalysed by recombinases (integrase, transposase, invertase) that are often found adjacent to the integration site of the phage genome, thus marking one of the prophage ends [40,41]. However, not all temperate phages encode recombinases. For example, filamentous vibriophage CTXφ exploits host-encoded recombinases XerC/D to integrate its genome into the bacterial chromosome [42]. We identified putative integrases in corticoviral elements of *Vibrio splendidus *12B01 (hit to integrase [YP_655517] of phage φMhaA1-PHL101, E = 9e-18), *Vibrionales *bacterium SWAT-3 (hit to integrase [AAO64735] of phage P2; E = 2e-74), and *Oceanobacter *sp. RED65 (hit to integrase [YP_654712] of phage F108; E = 3e-64). While putative prophages of *Vibrio *sp. MED222 and *Photobacterium profundum *SS9 contained fragments of putative transposase-coding genes (Fig. 1). In addition to an integrase, putative prophages of *Vibrio splendidus *12B01 and *Vibrionales *bacterium SWAT-3 encoded a CI/Cro-type repressor [43], similar to that of a λ-like bacteriophage DMS3 (accession no. YP_950425; E = 1e-13 and E = 7e-12, respectively). We do not currently know whether any of the PM2-like elements is inducible. However, the presence of recombinase-coding genes points to the mobile character of these elements and suggests that at least some of them are functional prophages.

### Ecological considerations

In order to determine in what ecological niches the putative PM2-like prophages could be traced, we checked the origins of the eleven bacteria harboring the corticoviral elements. All of them turned out to be residents of the aquatic ecosystem and were classified into the phylum *Proteobacteria *in the NCBI Taxonomy database [44].

Marine bacteriophages are the most abundant biological entities in the oceans. Since the oceans are the world's largest biosphere, marine phages are probably the most abundant biological entities on Earth, playing a vital role in the carbon cycling through marine food webs, gene transfer, and conversion of hosts by lysogeny ([45,46] and references therein). Over 5100 bacterial viruses have been examined in the electron microscope from 1959 to 2000. Of these, about 4950 phages (96.4%) were tailed (order *Caudovirales*) and only 186 phages (3.6%) were cubic, filamentous, or pleomorphic [47]. Tailed phage genomes were also observed to be in great abundance maintained inside the cell, constituting as much as 10 – 20% of some bacterial genomes (reviewed in [41]).

In order to compare the relative abundance of PM2-like elements maintained within aquatic bacteria with that of the tailed bacteriophages, 269 genomes of aquatic bacteria available in GenBank (01.05.2007; see Additional file 1) were searched for the presence of putative prophages of tailed viruses. For results to be comparable, the same identification approach as used here for the corticoviral elements was applied. We used the viral "self" determinants of the tailed phages (marine myoviruses [five species], podoviruses [four species], and siphoviruses [two species], as well as from well-studied enterobacterial phages P2 [*Myoviridae*], λ [*Siphoviridae*], and T7 [*Podoviridae*]), i.e. MCP and the larger subunit of the terminase protein, although other virion proteins were suggested to suite better for prophage detection [41]. If the bacterial genomes contained genes coding for both selected proteins, we assumed that a prophage is present on the chromosome (Table 1). As the tailed phages are not the object of this manuscript, no further detailed analysis was carried out. However, using this approach the abundance of the putative "tailed" prophages represents the maximal chance to be detected. The highest number of matches, twenty-two, was obtained with MCP and terminase protein sequences of enterophage P2. Among marine tailed phages analysed, the most abundant seems to be bacteriophage VHML-type, with close relatives identified in ten bacterial species. Enteric siphovirus λ and marine podovirus VP16C each had three relatives among 269 aquatic bacterial genomes analysed. Only one of these bacterial species (*Vibrio splendidus *12B01) contained both, the "self" determinants of the tailed phage and the putative PM2-like prophage (see Additional file 1). Notably, the two most abundant tailed phages, P2 and VHML, are temperate. Therefore, it seems that their abundance in the genomes of aquatic bacteria reflects the lifestyle they utilize. Interestingly, PM2 and VHML had almost the same number of matches (11 versus 10, respectively) (Table 1). However, it should be noted that PM2 is a strictly lytic phage, which has never been shown to enter the lysogenic pathway. There are no grounds to believe that integration frequency of lytic PM2-like phages should differ from that of the lytic tailed phages. Furthermore, we assume that the quantity of prophage-like elements is related to the quantity of free-living viruses.

**Table 1:** Abundance of prophage-like elements in 269 aquatic bacterial genomes

**Phage**	**Accession number**	**Virus family**	**MCP in dif. bacteria**	**Terminase in dif. bacteria**	**Matches**	References
PM2	NC_000867	Corticoviridae (L)^a^	11	11	11	[19]
T7	NC_001604	Podoviridae (L)	1	1	-	[59]
VP4	NC_007149	Podoviridae (?)	1	1	-	unpublished
P-SSP7	NC_006882	Podoviridae (T?)	1	1	-	[60]
VpV262	NC_003907	Podoviridae (L)	-	5	-	[61]
VP16C	AY328853	Podoviridae (T?)	2	24	2	[62]
P2	NC_001895	Myoviridae (T)	21	22	21	unpublished
S-PM2	NC_006820	Myoviridae (L)	-	13	-	[63]
KVP40	NC_005083	Myoviridae (L?)	-	5	-	[64]
VHML^b^	NC_004456	Myoviridae (T)	11	26	10	[65]
P-SSM2	NC_006883	Myoviridae (L)	-	14	-	[60]
P-SSM4	NC_006884	Myoviridae (L)	-	13	-	[60]
**λ**	NC_001416	Siphoviridae (T)	3	25	3	[66]
φHSIC	NC_006953	Siphoviridae (P)	3	4	-	[67]
φJL001	NC_006938	Siphoviridae (P)	2	6	-	[68]

^a ^– letter in the parenthesis denotes whether bacteriophage is lytic (L), temperate (T) or pseudo-temperate (P).

^b ^– VHML putative coat protein contains abundant peptidase domain (pfam01343) [65].

Therefore, only homologs for which global sequence alignments with the major coat protein were possible were further analyzed.

PM2 is the only icoshedral tailless marine bacteriophage characterized in detail. However, a number of transmission electron microscopy (TEM) studies have showed that tailless or short-tailed phages are the predominant morphotype in marine samples [48-52]. Since 96% of phages were proposed to be tailed [47], this observation was explained as a result of the dissociation of tails from the capsids during the sample preparation for TEM [46]. Our data presented here suggests that the abundance of PM2-like viruses versus tailed phages in the aquatic ecosystem (Table 1) might be vastly underestimated (using a prophage frequency criterion and assuming that mutational rate of the tailed and the PM2-like prophages is the same). This is also supported by the recent sampling of bacteriophages in alkaline hot springs at different geographical locations. Out of one-hundred-fifteen bacteriophage strains isolated during a trial, 50% were icosahedral tailless dsDNA phages, while tailed ones constituted only 44% of isolates [53], suggesting that tailed phages might not be dominant species in all ecological niches. On the other hand, the recent metagenomic study on free-living marine viruses suggested that the most dominant phages are those related to tailed cyanophage P-SSM2 [54]. However, the abundance of P-SSM2-like phages is questionable as more than 91% of the metagenomic sequences were not significantly similar to any of those in the database [54], therefore leaving space for alternative interpretations when more virus sequence data will be available. Interestingly, the same metagenomic study revealed the previously overlooked high abundance of small icosahedral tailless ssDNA phages (*Microviridae *virus family) in the samples obtained from the Sargasso Sea [54]. It is clear that the relative abundances of any type of viruses cannot be accurately accessed until much more data is available. It is also obviously that phage ecology is only gradually releasing its secrets and further surprises are anticipated.

## Conclusion

To our knowledge this is the first study showing that elements related to tailless dsDNA bacteriophages can be identified in bacterial genomes. The comparative analysis of the newly identified thirteen putative PM2-like elements, allowed us to test the innate viral "self" hypothesis [5,6,8,13]. We show here that the previously proposed most conserved viral "self" determinants, the major coat protein and the packaging ATPase, were the only proteins that could be found in all detected corticoviral elements. We also argue here that the genes needed for viral genome replication, as well as for host cell lysis, belong to the "nonself" category of genes, and can be acquired via horizontal gene exchange as needed. Therefore, proteins of the "nonself" category should not be used for the phylogenetic comparisons of different bacteriophages. In general, we believe that all viral proteins that are in one way or another involved in the viral interaction with its host belong to the "nonself" category. Furthermore, we challenge here the common knowledge that the vast majority of bacteriophages in aquatic environments belong to the order *Caudovirales *(dsDNA phages with tails). We suggest that abundance of PM2-like viruses in the aquatic environment as well as their importance in the ecology of aquatic microorganisms might be well underestimated.

## Methods

Putative corticoviral prophages were identified by homology-based searches against the nonredundant protein database at NCBI. The major coat protein P2 (accession no. NP_049903) of bacteriophage PM2 was used as a query in the PSI-BLAST [55] searches with the default parameters (BLOSUM62 matrix, 0.005 as an E-value cutoff). The search was iterated until no new sequences were found above the 0.005 threshold value (two iterations). In the case of a positive hit a ~15 kb region around the gene coding for the counterpart of the phage PM2 protein P2 was searched for putative prophage sequences using Vector NTI Suite 8.0 (InforMax Inc). It should be noted that outside the selected 15 kb region cellular genes (involved in cellular metabolism or abundant in other bacteria in which putative prophages could not be detected) started to appear outside that region. Open reading frames (ORF) were assigned as PM2-like genes if 1) the ORF pattern resembled that of PM2, 2) their putative protein products were clearly recognizable (up to 47% identity observed), 3) could be related to PM2 proteins by PSI-BLAST searches either directly, or through PM2-like gene products identified in other putative prophages. In the case of lower similarity (cutoff of 16% identity) other comparisons such as secondary structure [56] and protein topology [57] were used to confirm the relationship between the protein sequences. Major coat protein homology detection and structure prediction was carried out using the HHpred program with the default parameters [58]. Nucleotide coordinates of the corticoviral elements detected in this study can be found in the supplemental material table (see Additional file 1).

## Authors' contributions

MK collected, analyzed and interpreted the data, and drafted the manuscript. DHB interpreted the data and revised the manuscript. All authors read and approved the final manuscript

## Supplementary Material

Additional file 1 Table S1. Characteristics of the aquatic bacterial species analyzed in this study. The table provides information on the 269 aquatic bacterial species analyzed in this study for the presence of the putative prophages of tailed bacterial viruses. In addition, exact nucleotide coordinates of the putative corticoviral elements identified in this study are indicated. Click here for file
