# Supplementary material for: Putative prophages related to lytic tailless marine dsDNA phage PM2 are widespread in the genomes of aquatic bacteria
Source: BMC Genomics. 2007 Jul 16;8:236. doi: 10.1186/1471-2164-8-236 (PMC1950889; doi:10.1186/1471-2164-8-236)
Supplement: Additional file 1 — Table S1. Characteristics of the aquatic bacterial species analyzed in this study. The table provides information on the 269 aquatic bacterial species analyzed in this study for the presence of the putative prophages of tailed bacterial viruses. In addition, exact nucleotide coordinates of the putative corticoviral elements identified in this study are indicated. [file 1471-2164-8-236-S1.doc]

Table S1. Characteristics of the aquatic bacterial species analyzed in this study

| **Nr.** | Organism | **Chromo-some** | **Accession number** | **Genome size** | **Phylum; subphylum^b^** | **Nucleotide coordinates of the putative corticoviral prophages^c^** | **Distribution of the “self” determinants of tailed phages in the aquatic bacteria analyzed** | |
| --- | --- | --- | --- | --- | --- | --- | --- | --- |
|  | Species with complete genomic sequences |  |  |  |  |  | Major capsid protein | Terminase (large subunit) |
|  | [Acidothermus cellulolyticus 11B](http://www.ncbi.nlm.nih.gov/Taxonomy/Browser/wwwtax.cgi?id=351607) |  | [NC_008578](http://www.ncbi.nlm.nih.gov/entrez/query.fcgi?db=genome&cmd=Retrieve&dopt=Overview&list_uids=20069) | 244354 bp | Actinobacteria |  |  |  |
|  | [Acinetobacter sp. ADP1](http://www.ncbi.nlm.nih.gov/Taxonomy/Browser/wwwtax.cgi?id=62977) |  | [NC_005966](http://www.ncbi.nlm.nih.gov/genomes/framik.cgi?db=Genome&gi=411) | 3598621 bp | Proteobacteria; Gammaproteobacteria |  |  |  |
|  | [Aeromonas hydrophila subsp. hydrophila ATCC 7966](http://www.ncbi.nlm.nih.gov/Taxonomy/Browser/wwwtax.cgi?id=380703) |  | [NC_008570](http://www.ncbi.nlm.nih.gov/genomes/framik.cgi?db=Genome&gi=20061) | 4744448 bp | Proteobacteria; Gammaproteobacteria |  |  | S-PM2, P-SSM2, P-SSM4 |
|  | [Aeromonas salmonicida subsp. salmonicida A449](http://www.ncbi.nlm.nih.gov/Taxonomy/Browser/wwwtax.cgi?id=382245) |  | [NC_009348](http://www.ncbi.nlm.nih.gov/genomes/framik.cgi?db=Genome&gi=20863) | 4702402 bp | Proteobacteria; Gammaproteobacteria |  | P2 | P2 |
|  | [Alcanivorax borkumensis SK2](http://www.ncbi.nlm.nih.gov/Taxonomy/Browser/wwwtax.cgi?id=393595) |  | [NC_008260](http://www.ncbi.nlm.nih.gov/genomes/framik.cgi?db=Genome&gi=19683) | 3120143 bp | Proteobacteria; Gammaproteobacteria |  |  |  |
|  | [Alkalilimnicola ehrlichei MLHE-1](http://www.ncbi.nlm.nih.gov/Taxonomy/Browser/wwwtax.cgi?id=187272) |  | [NC_008340](http://www.ncbi.nlm.nih.gov/entrez/query.fcgi?db=genome&cmd=Retrieve&dopt=Overview&list_uids=19763) | 3275944 bp | Proteobacteria; Gammaproteobacteria |  |  |  |
|  | [Anabaena variabilis ATCC 29413](http://www.ncbi.nlm.nih.gov/Taxonomy/Browser/wwwtax.cgi?id=240292) |  | [NC_007413](http://www.ncbi.nlm.nih.gov/genomes/framik.cgi?db=Genome&gi=18763) | 6365727 bp | Cyanobacteria |  |  |  |
|  | [Aquifex aeolicus VF5^a^](http://www.ncbi.nlm.nih.gov/Taxonomy/Browser/wwwtax.cgi?id=224324) |  | [NC_000918](http://www.ncbi.nlm.nih.gov/genomes/framik.cgi?db=Genome&gi=133) | 1551335 bp | Aquificae |  |  |  |
|  | [Bdellovibrio bacteriovorus HD100](http://www.ncbi.nlm.nih.gov/Taxonomy/Browser/wwwtax.cgi?id=264462) |  | [NC_005363](http://www.ncbi.nlm.nih.gov/genomes/framik.cgi?db=Genome&gi=384) | 3782950 bp | Proteobacteria; Deltaproteobacteria |  |  |  |
|  | [Candidatus Pelagibacter ubique HTCC1062](http://www.ncbi.nlm.nih.gov/Taxonomy/Browser/wwwtax.cgi?id=335992) |  | [NC_007205](http://www.ncbi.nlm.nih.gov/genomes/framik.cgi?db=Genome&gi=18526) | 1308759 bp | Proteobacteria; Alphaproteobacteria |  |  |  |
|  | [Candidatus Protochlamydia amoebophila UWE25](http://www.ncbi.nlm.nih.gov/Taxonomy/Browser/wwwtax.cgi?id=264201) |  | [NC_005861](http://www.ncbi.nlm.nih.gov/genomes/framik.cgi?db=Genome&gi=399) | 2414465 bp | Chlamydiae/Verrucomicrobia group |  |  |  |
|  | [Candidatus Ruthia magnifica str. Cm (Calyptogena magnifica)](http://www.ncbi.nlm.nih.gov/Taxonomy/Browser/wwwtax.cgi?id=413404) |  | [NC_008610](http://www.ncbi.nlm.nih.gov/genomes/framik.cgi?db=Genome&gi=20101) | 1160782 bp | Proteobacteria; Gammaproteobacteria |  |  |  |
|  | [Carboxydothermus hydrogenoformans Z-2901](http://www.ncbi.nlm.nih.gov/Taxonomy/Browser/wwwtax.cgi?id=246194) |  | [NC_007503](http://www.ncbi.nlm.nih.gov/genomes/framik.cgi?db=Genome&gi=18853) | 2401520 bp | Firmicutes |  | φJL001 | φHSIC |
|  | [Caulobacter crescentus CB15](http://www.ncbi.nlm.nih.gov/Taxonomy/Browser/wwwtax.cgi?id=190650) |  | [NC_002696](http://www.ncbi.nlm.nih.gov/genomes/framik.cgi?db=Genome&gi=177) | 4016947 bp | Proteobacteria; Alphaproteobacteria |  |  |  |
|  | [Chlorobium chlorochromatii CaD3](http://www.ncbi.nlm.nih.gov/Taxonomy/Browser/wwwtax.cgi?id=340177) |  | [NC_007514](http://www.ncbi.nlm.nih.gov/genomes/framik.cgi?db=Genome&gi=18909) | 2572079 bp | Bacteroidetes/Chlorobi group |  |  |  |
|  | [Chlorobium phaeobacteroides DSM 266](http://www.ncbi.nlm.nih.gov/Taxonomy/Browser/wwwtax.cgi?id=290317) |  | [NC_008639](http://www.ncbi.nlm.nih.gov/entrez/query.fcgi?db=genome&cmd=Retrieve&dopt=Overview&list_uids=20130) | 3133902 bp | Bacteroidetes/Chlorobi group |  |  |  |
|  | [Chlorobium tepidum TLS](http://www.ncbi.nlm.nih.gov/Taxonomy/Browser/wwwtax.cgi?id=194439) |  | [NC_002932](http://www.ncbi.nlm.nih.gov/genomes/framik.cgi?db=Genome&gi=247) | 2154946 bp | Bacteroidetes/Chlorobi group |  |  |  |
|  | [Chromobacterium violaceum ATCC 12472](http://www.ncbi.nlm.nih.gov/Taxonomy/Browser/wwwtax.cgi?id=243365) |  | [NC_005085](http://www.ncbi.nlm.nih.gov/genomes/framik.cgi?db=Genome&gi=321) | 4751080 bp | Proteobacteria; Betaproteobacteria |  | P2 |  |
|  | [Chromohalobacter salexigens DSM 3043](http://www.ncbi.nlm.nih.gov/Taxonomy/Browser/wwwtax.cgi?id=290398) |  | [NC_007963](http://www.ncbi.nlm.nih.gov/genomes/framik.cgi?db=Genome&gi=19382) | 3696649 bp | Proteobacteria; Gammaproteobacteria |  | P2 | P2 |
|  | [Colwellia psychrerythraea 34H](http://www.ncbi.nlm.nih.gov/Taxonomy/Browser/wwwtax.cgi?id=167879) |  | [NC_003910](http://www.ncbi.nlm.nih.gov/genomes/framik.cgi?db=Genome&gi=18527) | 5373180 bp | Proteobacteria; Gammaproteobacteria |  |  |  |
|  | [Cytophaga hutchinsonii ATCC 33406](http://www.ncbi.nlm.nih.gov/Taxonomy/Browser/wwwtax.cgi?id=269798) |  | [NC_008255](http://www.ncbi.nlm.nih.gov/genomes/framik.cgi?db=Genome&gi=19678) | 4433218 bp | Bacteroidetes/Chlorobi group |  |  |  |
|  | [Dechloromonas aromatica RCB](http://www.ncbi.nlm.nih.gov/Taxonomy/Browser/wwwtax.cgi?id=159087) |  | [NC_007298](http://www.ncbi.nlm.nih.gov/genomes/framik.cgi?db=Genome&gi=18621) | 4501104 bp | Proteobacteria; Betaproteobacteria |  |  |  |
|  | [Dehalococcoides sp. CBDB1](http://www.ncbi.nlm.nih.gov/Taxonomy/Browser/wwwtax.cgi?id=255470) |  | [NC_007356](http://www.ncbi.nlm.nih.gov/genomes/framik.cgi?db=Genome&gi=18679) | 1395502 bp | Chloroflexi |  |  |  |
|  | [Deinococcus geothermalis DSM 11300](http://www.ncbi.nlm.nih.gov/Taxonomy/Browser/wwwtax.cgi?id=319795) |  | [NC_008025](http://www.ncbi.nlm.nih.gov/genomes/framik.cgi?db=Genome&gi=19444) | 2467205 bp | Deinococcus-Thermus |  |  |  |
|  | [Desulfotalea psychrophila LSv54](http://www.ncbi.nlm.nih.gov/Taxonomy/Browser/wwwtax.cgi?id=177439) |  | [NC_006138](http://www.ncbi.nlm.nih.gov/genomes/framik.cgi?db=Genome&gi=490) | 3523383 bp | Proteobacteria; Deltaproteobacteria |  | VHML | VHML, P-SSM2 |
|  | [Desulfotomaculum reducens MI-1](http://www.ncbi.nlm.nih.gov/Taxonomy/Browser/wwwtax.cgi?id=349161) |  | [NC_009253](http://www.ncbi.nlm.nih.gov/entrez/query.fcgi?db=genome&cmd=Retrieve&dopt=Overview&list_uids=20749) | 3608104 bp | Firmicutes |  | VP16C, λ | VP16C, S-PM2, KVP40, VHML, P-SSM2, P-SSM4, λ |
|  | [Erythrobacter litoralis HTCC2594](http://www.ncbi.nlm.nih.gov/Taxonomy/Browser/wwwtax.cgi?id=314225) |  | [NC_007722](http://www.ncbi.nlm.nih.gov/genomes/framik.cgi?db=Genome&gi=19139) | 3052398 bp | Proteobacteria; Alphaproteobacteria |  |  |  |
|  | [Geobacillus kaustophilus HTA426](http://www.ncbi.nlm.nih.gov/Taxonomy/Browser/wwwtax.cgi?id=235909) |  | [NC_006510](http://www.ncbi.nlm.nih.gov/genomes/framik.cgi?db=Genome&gi=556) | 3544776 bp | Firmicutes |  |  |  |
|  | [Geobacillus thermodenitrificans NG80-2](http://www.ncbi.nlm.nih.gov/Taxonomy/Browser/wwwtax.cgi?id=420246) |  | [NC_009328](http://www.ncbi.nlm.nih.gov/genomes/framik.cgi?db=Genome&gi=20824) | 3550319 bp | Firmicutes |  |  |  |
|  | [Geobacter metallireducens GS-15](http://www.ncbi.nlm.nih.gov/Taxonomy/Browser/wwwtax.cgi?id=269799) |  | [NC_007517](http://www.ncbi.nlm.nih.gov/genomes/framik.cgi?db=Genome&gi=18912) | 3997420 bp | Proteobacteria; Deltaproteobacteria |  |  |  |
|  | [Gloeobacter violaceus PCC 7421](http://www.ncbi.nlm.nih.gov/entrez/query.fcgi?db=genomeprj&cmd=Retrieve&dopt=Overview&list_uids=9606) |  | [NC_005125](http://www.ncbi.nlm.nih.gov/entrez/query.fcgi?db=genome&cmd=Retrieve&dopt=Overview&list_uids=346) | 4659020 bp | Cyanobacteria |  |  |  |
|  | [Gramella forsetii KT0803](http://www.ncbi.nlm.nih.gov/Taxonomy/Browser/wwwtax.cgi?id=411154) |  | [NC_008571](http://www.ncbi.nlm.nih.gov/genomes/framik.cgi?db=Genome&gi=20062) | 3798465 bp | Bacteroidetes/Chlorobi group |  |  | φJL001 |
|  | [Hahella chejuensis KCTC 2396](http://www.ncbi.nlm.nih.gov/Taxonomy/Browser/wwwtax.cgi?id=349521) |  | [NC_007645](http://www.ncbi.nlm.nih.gov/genomes/framik.cgi?db=Genome&gi=19061) | 7215267 bp | Proteobacteria; Gammaproteobacteria |  | VP16C, P2, VHML, λ | VP16C, P2, VHML, λ |
|  | [Halorhodospira halophila SL1](http://www.ncbi.nlm.nih.gov/Taxonomy/Browser/wwwtax.cgi?id=349124) |  | [NC_008789](http://www.ncbi.nlm.nih.gov/entrez/query.fcgi?db=genome&cmd=Retrieve&dopt=Overview&list_uids=20280) | 2678452 bp | Proteobacteria; Gammaproteobacteria |  |  |  |
|  | [Herminiimonas arsenicoxydans](http://www.ncbi.nlm.nih.gov/Taxonomy/Browser/wwwtax.cgi?id=204773) |  | [NC_009138](http://www.ncbi.nlm.nih.gov/genomes/framik.cgi?db=Genome&gi=20634) | 3424307 bp | Proteobacteria; Betaproteobacteria |  |  | φJL001 |
|  | [Hyphomonas neptunium ATCC 15444](http://www.ncbi.nlm.nih.gov/Taxonomy/Browser/wwwtax.cgi?id=228405) |  | [NC_008358](http://www.ncbi.nlm.nih.gov/genomes/framik.cgi?db=Genome&gi=19808) | 3705021 bp | Proteobacteria; Alphaproteobacteria |  |  |  |
|  | [Jannaschia sp. CCS1](http://www.ncbi.nlm.nih.gov/Taxonomy/Browser/wwwtax.cgi?id=290400) |  | [NC_007802](http://www.ncbi.nlm.nih.gov/genomes/framik.cgi?db=Genome&gi=19220) | 4317977 bp | Proteobacteria; Alphaproteobacteria |  |  |  |
|  | [Magnetococcus sp. MC-1](http://www.ncbi.nlm.nih.gov/Taxonomy/Browser/wwwtax.cgi?id=156889) |  | [NC_008576](http://www.ncbi.nlm.nih.gov/entrez/query.fcgi?db=genome&cmd=Retrieve&dopt=Overview&list_uids=20067) | 4719581 bp | Proteobacteria; unclassified |  | VHML | VP16C, VHML, λ |
|  | [Magnetospirillum magneticum AMB-1](http://www.ncbi.nlm.nih.gov/Taxonomy/Browser/wwwtax.cgi?id=342108) |  | [NC_007626](http://www.ncbi.nlm.nih.gov/genomes/framik.cgi?db=Genome&gi=19021) | 4967148 bp | Proteobacteria; Alphaproteobacteria |  | VHML | T7, VP4, P-SSP7, VpV262, VP16C, VHML, λ |
|  | [Maricaulis maris MCS10](http://www.ncbi.nlm.nih.gov/Taxonomy/Browser/wwwtax.cgi?id=394221) |  | [NC_008347](http://www.ncbi.nlm.nih.gov/entrez/query.fcgi?db=genome&cmd=Retrieve&dopt=Overview&list_uids=19797) | 3368780 bp | Proteobacteria; Alphaproteobacteria |  |  |  |
|  | [Marinobacter aquaeolei VT8](http://www.ncbi.nlm.nih.gov/Taxonomy/Browser/wwwtax.cgi?id=351348) |  | [NC_008740](http://www.ncbi.nlm.nih.gov/entrez/query.fcgi?db=genome&cmd=Retrieve&dopt=Overview&list_uids=20231) | 4326849 bp | Proteobacteria; Gammaproteobacteria |  |  |  |
|  | [Mesorhizobium sp. BNC1](http://www.ncbi.nlm.nih.gov/Taxonomy/Browser/wwwtax.cgi?id=266779) |  | [NC_008254](http://www.ncbi.nlm.nih.gov/genomes/framik.cgi?db=Genome&gi=19677) | 4412446 bp | Proteobacteria; Alphaproteobacteria |  |  |  |
|  | [**Methylibium petroleiphilum PM1**](http://www.ncbi.nlm.nih.gov/Taxonomy/Browser/wwwtax.cgi?mode=Info&id=420662&lvl=3&lin=f&keep=1&srchmode=1&unlock)**^a^** |  | [**NC_008825**](http://www.ncbi.nlm.nih.gov/entrez/query.fcgi?db=genome&cmd=Retrieve&dopt=Overview&list_uids=20316) | **4044195 bp** | **Proteobacteria; Betaproteobacteria** | **1972178-1985805** |  |  |
|  | [**Methylobacillus flagellatus KT**](http://www.ncbi.nlm.nih.gov/Taxonomy/Browser/wwwtax.cgi?id=265072) |  | [**NC_007947**](http://www.ncbi.nlm.nih.gov/genomes/framik.cgi?db=Genome&gi=19366) | **2971517 bp** | **Proteobacteria; Betaproteobacteria** | **1 (866351-875339), 2 (1009383-1018371)** |  |  |
|  | [Moorella thermoacetica ATCC 39073](http://www.ncbi.nlm.nih.gov/Taxonomy/Browser/wwwtax.cgi?id=264732) |  | [NC_007644](http://www.ncbi.nlm.nih.gov/genomes/framik.cgi?db=Genome&gi=19060) | 2628784 bp | Firmicutes |  |  |  |
|  | [Mycobacterium vanbaalenii PYR-1](http://www.ncbi.nlm.nih.gov/Taxonomy/Browser/wwwtax.cgi?id=350058) |  | [NC_008726](http://www.ncbi.nlm.nih.gov/entrez/query.fcgi?db=genome&cmd=Retrieve&dopt=Overview&list_uids=20217) | 6491865 bp | Actinobacteria |  |  |  |
|  | [Mycoplasma mobile 163K](http://www.ncbi.nlm.nih.gov/Taxonomy/Browser/wwwtax.cgi?id=267748) |  | [NC_006908](http://www.ncbi.nlm.nih.gov/genomes/framik.cgi?db=Genome&gi=402) | 777079 bp | Firmicutes; Mollicutes |  |  |  |
|  | [Nitrosococcus oceani ATCC 19707](http://www.ncbi.nlm.nih.gov/Taxonomy/Browser/wwwtax.cgi?id=323261) |  | [NC_007484](http://www.ncbi.nlm.nih.gov/genomes/framik.cgi?db=Genome&gi=18834) | 3481691 bp | Proteobacteria; Gammaproteobacteria |  |  |  |
|  | [Nitrosomonas eutropha C91](http://www.ncbi.nlm.nih.gov/Taxonomy/Browser/wwwtax.cgi?id=335283) |  | [NC_008344](http://www.ncbi.nlm.nih.gov/entrez/query.fcgi?db=genome&cmd=Retrieve&dopt=Overview&list_uids=19767) | 2661057 bp | Proteobacteria; Betaproteobacteria |  |  |  |
|  | [Nocardioides sp. JS614](http://www.ncbi.nlm.nih.gov/Taxonomy/Browser/wwwtax.cgi?id=196162) |  | [NC_008699](http://www.ncbi.nlm.nih.gov/genomes/framik.cgi?db=Genome&gi=20190) | 4985871 bp | Actinobacteria |  |  |  |
|  | [Nostoc sp. PCC 7120](http://www.ncbi.nlm.nih.gov/entrez/query.fcgi?db=genomeprj&cmd=Retrieve&dopt=Overview&list_uids=244) |  | [NC_003272](http://www.ncbi.nlm.nih.gov/entrez/query.fcgi?db=genome&cmd=Retrieve&dopt=Overview&list_uids=217) | 6413770 bp | Cyanobacteria |  |  |  |
|  | [Oceanobacillus iheyensis HTE831](http://www.ncbi.nlm.nih.gov/Taxonomy/Browser/wwwtax.cgi?id=221109) |  | [NC_004193](http://www.ncbi.nlm.nih.gov/genomes/framik.cgi?db=Genome&gi=253) | 3630528 bp | Firmicutes |  |  |  |
|  | [Oenococcus oeni PSU-1](http://www.ncbi.nlm.nih.gov/Taxonomy/Browser/wwwtax.cgi?id=203123) |  | [NC_008528](http://www.ncbi.nlm.nih.gov/genomes/framik.cgi?db=Genome&gi=20018) | 1780517 bp | Firmicutes |  |  |  |
|  | [Pelobacter carbinolicus DSM 2380](http://www.ncbi.nlm.nih.gov/Taxonomy/Browser/wwwtax.cgi?id=338963) |  | [NC_007498](http://www.ncbi.nlm.nih.gov/genomes/framik.cgi?db=Genome&gi=18848) | 3665893 bp | Proteobacteria; Deltaproteobacteria |  | VHML |  |
|  | [Pelobacter propionicus DSM 2379](http://www.ncbi.nlm.nih.gov/Taxonomy/Browser/wwwtax.cgi?id=338966) |  | [NC_008609](http://www.ncbi.nlm.nih.gov/entrez/query.fcgi?db=genome&cmd=Retrieve&dopt=Overview&list_uids=20100) | 4008000 bp | Proteobacteria; Deltaproteobacteria |  |  |  |
|  | [Pelodictyon luteolum DSM 273](http://www.ncbi.nlm.nih.gov/Taxonomy/Browser/wwwtax.cgi?id=319225) |  | [NC_007512](http://www.ncbi.nlm.nih.gov/genomes/framik.cgi?db=Genome&gi=18907) | 2364842 bp | Bacteroidetes/Chlorobi group |  |  |  |
|  | [Photobacterium profundum SS9](http://www.ncbi.nlm.nih.gov/Taxonomy/Browser/wwwtax.cgi?id=298386) | 1 | [NC_006370](http://www.ncbi.nlm.nih.gov/genomes/framik.cgi?db=Genome&gi=512) | 4085304 bp | Proteobacteria; Gammaproteobacteria |  |  |  |
|  | [**Photobacterium profundum SS9**](http://www.ncbi.nlm.nih.gov/Taxonomy/Browser/wwwtax.cgi?id=298386) | **2** | [**NC_006371**](http://www.ncbi.nlm.nih.gov/genomes/framik.cgi?db=Genome&gi=513) | **2237943 bp** | **Proteobacteria; Gammaproteobacteria** | **1074738-1089177** |  |  |
|  | [Polaromonas naphthalenivorans CJ2](http://www.ncbi.nlm.nih.gov/Taxonomy/Browser/wwwtax.cgi?id=365044) |  | [NC_008781](http://www.ncbi.nlm.nih.gov/entrez/query.fcgi?db=genome&cmd=Retrieve&dopt=Overview&list_uids=20272) | 4410291 bp | Proteobacteria; Betaproteobacteria |  | P2 | VP16C, P2, λ |
|  | [Polaromonas sp. JS666](http://www.ncbi.nlm.nih.gov/Taxonomy/Browser/wwwtax.cgi?id=296591) |  | [NC_007948](http://www.ncbi.nlm.nih.gov/genomes/framik.cgi?db=Genome&gi=19367) | 5200264 bp | Proteobacteria; Betaproteobacteria |  |  |  |
|  | [Polynucleobacter sp. QLW-P1DMWA-1](http://www.ncbi.nlm.nih.gov/Taxonomy/Browser/wwwtax.cgi?id=312153) |  | [NC_009379](http://www.ncbi.nlm.nih.gov/genomes/framik.cgi?db=Genome&gi=20894) | 2159490 bp | Proteobacteria; Betaproteobacteria |  |  | φJL001 |
|  | [Prochlorococcus marinus str. AS9601](http://www.ncbi.nlm.nih.gov/Taxonomy/Browser/wwwtax.cgi?id=146891) |  | [NC_008816](http://www.ncbi.nlm.nih.gov/genomes/framik.cgi?db=Genome&gi=20307) | 1669886 bp | Cyanobacteria |  |  |  |
|  | [Prochlorococcus marinus str. MIT 9301](http://www.ncbi.nlm.nih.gov/Taxonomy/Browser/wwwtax.cgi?id=167546) |  | [NC_009091](http://www.ncbi.nlm.nih.gov/genomes/framik.cgi?db=Genome&gi=20587) | 1641879 bp | Cyanobacteria |  |  |  |
|  | [Prochlorococcus marinus str. MIT 9303](http://www.ncbi.nlm.nih.gov/Taxonomy/Browser/wwwtax.cgi?id=59922) |  | [NC_008820](http://www.ncbi.nlm.nih.gov/genomes/framik.cgi?db=Genome&gi=20311) | 2682675 bp | Cyanobacteria |  |  |  |
|  | [Prochlorococcus marinus str. MIT 9312](http://www.ncbi.nlm.nih.gov/Taxonomy/Browser/wwwtax.cgi?id=74546) |  | [NC_007577](http://www.ncbi.nlm.nih.gov/genomes/framik.cgi?db=Genome&gi=18972) | 1709204 bp | Cyanobacteria |  |  |  |
|  | [Prochlorococcus marinus str. MIT 9313](http://www.ncbi.nlm.nih.gov/Taxonomy/Browser/wwwtax.cgi?id=74547) |  | [NC_005071](http://www.ncbi.nlm.nih.gov/genomes/framik.cgi?db=Genome&gi=319) | 2410873 bp | Cyanobacteria |  |  |  |
|  | [Prochlorococcus marinus str. MIT 9515](http://www.ncbi.nlm.nih.gov/Taxonomy/Browser/wwwtax.cgi?id=167542) |  | [NC_008817](http://www.ncbi.nlm.nih.gov/genomes/framik.cgi?db=Genome&gi=20308) | 1704176 bp | Cyanobacteria |  |  |  |
|  | [Prochlorococcus marinus str. NATL1A](http://www.ncbi.nlm.nih.gov/Taxonomy/Browser/wwwtax.cgi?id=167555) |  | [NC_008819](http://www.ncbi.nlm.nih.gov/genomes/framik.cgi?db=Genome&gi=20310) | 1864731 bp | Cyanobacteria |  |  |  |
|  | [Prochlorococcus marinus str. NATL2A](http://www.ncbi.nlm.nih.gov/Taxonomy/Browser/wwwtax.cgi?id=59920) |  | [NC_007335](http://www.ncbi.nlm.nih.gov/genomes/framik.cgi?db=Genome&gi=18655) | 1842899 bp | Cyanobacteria |  |  |  |
|  | [Prochlorococcus marinus subsp. marinus str. CCMP1375](http://www.ncbi.nlm.nih.gov/Taxonomy/Browser/wwwtax.cgi?id=167539) |  | [NC_005042](http://www.ncbi.nlm.nih.gov/genomes/framik.cgi?db=Genome&gi=310) | 1751080 bp | Cyanobacteria |  |  |  |
|  | [Prochlorococcus marinus subsp. pastoris str. CCMP1986](http://www.ncbi.nlm.nih.gov/Taxonomy/Browser/wwwtax.cgi?id=59919) |  | [NC_005072](http://www.ncbi.nlm.nih.gov/genomes/framik.cgi?db=Genome&gi=318) | 1657990 bp | Cyanobacteria |  |  |  |
|  | [Prosthecochloris vibrioformis DSM 265](http://www.ncbi.nlm.nih.gov/Taxonomy/Browser/wwwtax.cgi?id=290318) |  | [NC_009337](http://www.ncbi.nlm.nih.gov/entrez/query.fcgi?db=genome&cmd=Retrieve&dopt=Overview&list_uids=20852) | 1966858 bp | Bacteroidetes/Chlorobi group |  |  |  |
|  | [Prosthecochloris vibrioformis DSM 265](http://www.ncbi.nlm.nih.gov/Taxonomy/Browser/wwwtax.cgi?id=290318) |  | [NC_009337](http://www.ncbi.nlm.nih.gov/genomes/framik.cgi?db=Genome&gi=20852) | 1966858 bp | Bacteroidetes/Chlorobi group |  |  |  |
|  | [Pseudoalteromonas atlantica T6c](http://www.ncbi.nlm.nih.gov/Taxonomy/Browser/wwwtax.cgi?id=342610) |  | [NC_008228](http://www.ncbi.nlm.nih.gov/genomes/framik.cgi?db=Genome&gi=19650) | 5187005 bp | Proteobacteria; Gammaproteobacteria |  | P2 | P2 |
|  | [Pseudoalteromonas haloplanktis TAC125](http://www.ncbi.nlm.nih.gov/Taxonomy/Browser/wwwtax.cgi?id=326442) | 1 | [NC_007481](http://www.ncbi.nlm.nih.gov/genomes/framik.cgi?db=Genome&gi=18831) | 3214944 bp | Proteobacteria; Gammaproteobacteria |  |  |  |
|  | [Pseudoalteromonas haloplanktis TAC125](http://www.ncbi.nlm.nih.gov/Taxonomy/Browser/wwwtax.cgi?id=326442) | 2 | [NC_007482](http://www.ncbi.nlm.nih.gov/genomes/framik.cgi?db=Genome&gi=18832) | 635328 bp | Proteobacteria; Gammaproteobacteria |  |  |  |
|  | [Pseudomonas fluorescens Pf-5](http://www.ncbi.nlm.nih.gov/Taxonomy/Browser/wwwtax.cgi?id=220664) |  | [NC_004129](http://www.ncbi.nlm.nih.gov/genomes/framik.cgi?db=Genome&gi=18509) | 7074893 bp | Proteobacteria; Gammaproteobacteria |  | φHSIC | VP16C, VHML, λ |
|  | [Psychromonas ingrahamii 37](http://www.ncbi.nlm.nih.gov/Taxonomy/Browser/wwwtax.cgi?id=357804) |  | [NC_008709](http://www.ncbi.nlm.nih.gov/entrez/query.fcgi?db=genome&cmd=Retrieve&dopt=Overview&list_uids=20200) | 4559598 bp | Proteobacteria; Gammaproteobacteria |  |  |  |
|  | [Ralstonia eutropha H16](http://www.ncbi.nlm.nih.gov/Taxonomy/Browser/wwwtax.cgi?id=381666) | 1 | [NC_008313](http://www.ncbi.nlm.nih.gov/genomes/framik.cgi?db=Genome&gi=19736) | 4052032 bp | Proteobacteria; Betaproteobacteria |  |  |  |
|  | [**Ralstonia eutropha H16**](http://www.ncbi.nlm.nih.gov/Taxonomy/Browser/wwwtax.cgi?id=381666) | **2** | [**NC_008314**](http://www.ncbi.nlm.nih.gov/entrez/viewer.fcgi??db=nucleotide&val=NC_008314) | **2912490 bp** | **Proteobacteria; Betaproteobacteria** | **1462494-1476008** |  |  |
|  | [Ralstonia eutropha JMP134](http://www.ncbi.nlm.nih.gov/Taxonomy/Browser/wwwtax.cgi?id=264198) | 1 | [NC_007347](http://www.ncbi.nlm.nih.gov/genomes/framik.cgi?db=Genome&gi=18667) | 3806533 bp | Proteobacteria; Betaproteobacteria |  |  | VpV262 |
|  | [Ralstonia eutropha JMP134](http://www.ncbi.nlm.nih.gov/Taxonomy/Browser/wwwtax.cgi?id=264198) | 2 | [NC_007348](http://www.ncbi.nlm.nih.gov/genomes/framik.cgi?db=Genome&gi=18668) | 2726152 bp | Proteobacteria; Betaproteobacteria |  |  |  |
|  | [Ralstonia metallidurans CH34](http://www.ncbi.nlm.nih.gov/Taxonomy/Browser/wwwtax.cgi?id=266264) | 1 | [NC_007973](http://www.ncbi.nlm.nih.gov/genomes/framik.cgi?db=Genome&gi=19392) | 3928089 bp | Proteobacteria; Betaproteobacteria |  |  |  |
|  | [Ralstonia metallidurans CH34](http://www.ncbi.nlm.nih.gov/Taxonomy/Browser/wwwtax.cgi?id=266264) | 2 | [NC_007974](http://www.ncbi.nlm.nih.gov/genomes/framik.cgi?db=Genome&gi=19393) | 2580084 bp | Proteobacteria; Betaproteobacteria |  |  |  |
|  | [Rhodoferax ferrireducens T118](http://www.ncbi.nlm.nih.gov/Taxonomy/Browser/wwwtax.cgi?id=338969) |  | [NC_007908](http://www.ncbi.nlm.nih.gov/genomes/framik.cgi?db=Genome&gi=19327) | 4712337 bp | Proteobacteria; Betaproteobacteria |  |  |  |
|  | [Rhodopirellula baltica SH 1](http://www.ncbi.nlm.nih.gov/Taxonomy/Browser/wwwtax.cgi?id=243090) |  | [NC_005027](http://www.ncbi.nlm.nih.gov/genomes/framik.cgi?db=Genome&gi=308) | 7145576 bp | Planctomycetes |  |  |  |
|  | [Rhodopseudomonas palustris BisA53](http://www.ncbi.nlm.nih.gov/Taxonomy/Browser/wwwtax.cgi?id=316055) |  | [NC_008435](http://www.ncbi.nlm.nih.gov/entrez/query.fcgi?db=genome&cmd=Retrieve&dopt=Overview&list_uids=19886) | 5505494 bp | Proteobacteria; Alphaproteobacteria |  |  |  |
|  | [Rhodopseudomonas palustris BisB18](http://www.ncbi.nlm.nih.gov/Taxonomy/Browser/wwwtax.cgi?id=316056) |  | [NC_007925](http://www.ncbi.nlm.nih.gov/genomes/framik.cgi?db=Genome&gi=19344) | 5513844 bp | Proteobacteria; Alphaproteobacteria |  |  |  |
|  | [Rhodopseudomonas palustris BisB5](http://www.ncbi.nlm.nih.gov/Taxonomy/Browser/wwwtax.cgi?id=316057) |  | [NC_007958](http://www.ncbi.nlm.nih.gov/genomes/framik.cgi?db=Genome&gi=19377) | 4892717 bp | Proteobacteria; Alphaproteobacteria |  |  |  |
|  | [Rhodopseudomonas palustris CGA009](http://www.ncbi.nlm.nih.gov/Taxonomy/Browser/wwwtax.cgi?id=258594) |  | [NC_005296](http://www.ncbi.nlm.nih.gov/genomes/framik.cgi?db=Genome&gi=377) | 5459213 bp | Proteobacteria; Alphaproteobacteria |  |  |  |
|  | [Rhodopseudomonas palustris HaA2](http://www.ncbi.nlm.nih.gov/Taxonomy/Browser/wwwtax.cgi?id=316058) |  | [NC_007778](http://www.ncbi.nlm.nih.gov/genomes/framik.cgi?db=Genome&gi=19195) | 5331656 bp | Proteobacteria; Alphaproteobacteria |  |  |  |
|  | [Roseobacter denitrificans OCh 114](http://www.ncbi.nlm.nih.gov/Taxonomy/Browser/wwwtax.cgi?id=375451) |  | [NC_008209](http://www.ncbi.nlm.nih.gov/genomes/framik.cgi?db=Genome&gi=19631) | 4133097 bp | Proteobacteria; Alphaproteobacteria |  |  |  |
|  | [Saccharophagus degradans 2-40](http://www.ncbi.nlm.nih.gov/Taxonomy/Browser/wwwtax.cgi?id=203122) |  | [NC_007912](http://www.ncbi.nlm.nih.gov/genomes/framik.cgi?db=Genome&gi=19331) | 5057531 bp | Proteobacteria; Gammaproteobacteria |  |  |  |
|  | [Salinibacter ruber DSM 13855](http://www.ncbi.nlm.nih.gov/Taxonomy/Browser/wwwtax.cgi?id=309807) |  | [NC_007677](http://www.ncbi.nlm.nih.gov/genomes/framik.cgi?db=Genome&gi=19093) | 3551823 bp | Bacteroidetes/Chlorobi group |  |  |  |
|  | [Salinispora tropica CNB-440](http://www.ncbi.nlm.nih.gov/Taxonomy/Browser/wwwtax.cgi?id=369723) |  | [NC_009380](http://www.ncbi.nlm.nih.gov/genomes/framik.cgi?db=Genome&gi=20895) | 5183331 bp | Actinobacteria |  |  |  |
|  | [Shewanella amazonensis SB2B](http://www.ncbi.nlm.nih.gov/Taxonomy/Browser/wwwtax.cgi?id=326297) |  | [NC_008700](http://www.ncbi.nlm.nih.gov/entrez/query.fcgi?db=genome&cmd=Retrieve&dopt=Overview&list_uids=20191) | 4306142 bp | Proteobacteria; Gammaproteobacteria |  |  |  |
|  | [Shewanella baltica OS155](http://www.ncbi.nlm.nih.gov/Taxonomy/Browser/wwwtax.cgi?id=325240) |  | [NC_009052](http://www.ncbi.nlm.nih.gov/entrez/query.fcgi?db=genome&cmd=Retrieve&dopt=Overview&list_uids=20542) | 5127376 bp | Proteobacteria; Gammaproteobacteria |  | P2 | VP16C, P2, VHML, λ |
|  | [Shewanella denitrificans OS217](http://www.ncbi.nlm.nih.gov/Taxonomy/Browser/wwwtax.cgi?id=318161) |  | [NC_007954](http://www.ncbi.nlm.nih.gov/genomes/framik.cgi?db=Genome&gi=19373) | 4545906 bp | Proteobacteria; Gammaproteobacteria |  |  | VP16C, VHML, λ |
|  | [Shewanella frigidimarina NCIMB 400](http://www.ncbi.nlm.nih.gov/Taxonomy/Browser/wwwtax.cgi?id=318167) |  | [NC_008345](http://www.ncbi.nlm.nih.gov/entrez/query.fcgi?db=genome&cmd=Retrieve&dopt=Overview&list_uids=19795) | 4845257 bp | Proteobacteria; Gammaproteobacteria |  |  | VP16C, VHML, λ |
|  | [Shewanella loihica PV-4](http://www.ncbi.nlm.nih.gov/Taxonomy/Browser/wwwtax.cgi?id=323850) |  | [NC_009092](http://www.ncbi.nlm.nih.gov/genomes/framik.cgi?db=Genome&gi=20588) | 4602594 bp | Proteobacteria; Gammaproteobacteria |  |  |  |
|  | [Shewanella oneidensis MR-1](http://www.ncbi.nlm.nih.gov/Taxonomy/Browser/wwwtax.cgi?id=211586) |  | [NC_004347](http://www.ncbi.nlm.nih.gov/genomes/framik.cgi?db=Genome&gi=261) | 4969803 bp | Proteobacteria; Gammaproteobacteria |  |  |  |
|  | [Shewanella sp. ANA-3](http://www.ncbi.nlm.nih.gov/Taxonomy/Browser/wwwtax.cgi?id=94122) | 1 | [NC_008577](http://www.ncbi.nlm.nih.gov/entrez/query.fcgi?db=genome&cmd=Retrieve&dopt=Overview&list_uids=20068) | 4972204 bp | Proteobacteria; Gammaproteobacteria |  |  |  |
|  | [Shewanella sp. MR-4](http://www.ncbi.nlm.nih.gov/Taxonomy/Browser/wwwtax.cgi?id=60480) |  | [NC_008321](http://www.ncbi.nlm.nih.gov/entrez/query.fcgi?db=genome&cmd=Retrieve&dopt=Overview&list_uids=19744) | 4706287 bp | Proteobacteria; Gammaproteobacteria |  |  |  |
|  | [Shewanella sp. MR-7](http://www.ncbi.nlm.nih.gov/Taxonomy/Browser/wwwtax.cgi?id=60481) |  | [NC_008322](http://www.ncbi.nlm.nih.gov/entrez/query.fcgi?db=genome&cmd=Retrieve&dopt=Overview&list_uids=19745) | 4792610 bp | Proteobacteria; Gammaproteobacteria |  | P2 | P2 |
|  | [Shewanella sp. PV-4](http://www.ncbi.nlm.nih.gov/Taxonomy/Browser/wwwtax.cgi?id=323850) |  | [NC_009092](http://www.ncbi.nlm.nih.gov/entrez/query.fcgi?db=genome&cmd=Retrieve&dopt=Overview&list_uids=20588) | 4602594 bp | Proteobacteria; Gammaproteobacteria |  |  |  |
|  | [Shewanella sp. W3-18-1](http://www.ncbi.nlm.nih.gov/Taxonomy/Browser/wwwtax.cgi?id=351745) |  | [NC_008750](http://www.ncbi.nlm.nih.gov/entrez/query.fcgi?db=genome&cmd=Retrieve&dopt=Overview&list_uids=20241) | 4708380 bp | Proteobacteria; Gammaproteobacteria |  | P2 | P2 |
|  | [Silicibacter pomeroyi DSS-3](http://www.ncbi.nlm.nih.gov/Taxonomy/Browser/wwwtax.cgi?id=246200) |  | [NC_003911](http://www.ncbi.nlm.nih.gov/genomes/framik.cgi?db=Genome&gi=562) | 4109442 bp | Proteobacteria; Alphaproteobacteria |  |  |  |
|  | [Silicibacter sp. TM1040](http://www.ncbi.nlm.nih.gov/Taxonomy/Browser/wwwtax.cgi?id=292414) |  | [NC_008044](http://www.ncbi.nlm.nih.gov/genomes/framik.cgi?db=Genome&gi=19463) | 3200938 bp | Proteobacteria; Alphaproteobacteria |  | φHSIC | VP16C, VHML, λ |
|  | [Sphingopyxis alaskensis RB2256](http://www.ncbi.nlm.nih.gov/Taxonomy/Browser/wwwtax.cgi?id=317655) |  | [NC_008048](http://www.ncbi.nlm.nih.gov/genomes/framik.cgi?db=Genome&gi=19467) | 3345170 bp | Proteobacteria; Alphaproteobacteria |  |  |  |
|  | [Synechococcus elongatus PCC 6301](http://www.ncbi.nlm.nih.gov/Taxonomy/Browser/wwwtax.cgi?id=269084) |  | [NC_006576](http://www.ncbi.nlm.nih.gov/genomes/framik.cgi?db=Genome&gi=564) | 2696255 bp | Cyanobacteria |  |  | VP16C, VHML, λ |
|  | [Synechococcus elongatus PCC 7942](http://www.ncbi.nlm.nih.gov/Taxonomy/Browser/wwwtax.cgi?id=1140) |  | [NC_007604](http://www.ncbi.nlm.nih.gov/genomes/framik.cgi?db=Genome&gi=18999) | 2695903 bp | Cyanobacteria |  |  |  |
|  | [Synechococcus sp. CC9311](http://www.ncbi.nlm.nih.gov/entrez/query.fcgi?db=genomeprj&cmd=Retrieve&dopt=Overview&list_uids=12530) |  | [NC_008319](http://www.ncbi.nlm.nih.gov/entrez/query.fcgi?db=genome&cmd=Retrieve&dopt=Overview&list_uids=19742) | 2606750 bp | Cyanobacteria |  |  |  |
|  | [Synechococcus sp. CC9605](http://www.ncbi.nlm.nih.gov/Taxonomy/Browser/wwwtax.cgi?id=110662) |  | [NC_007516](http://www.ncbi.nlm.nih.gov/genomes/framik.cgi?db=Genome&gi=18911) | 2510659 bp | Cyanobacteria |  |  | φJL001 |
|  | [Synechococcus sp. CC9902](http://www.ncbi.nlm.nih.gov/Taxonomy/Browser/wwwtax.cgi?id=316279) |  | [NC_007513](http://www.ncbi.nlm.nih.gov/genomes/framik.cgi?db=Genome&gi=18908) | 2234828 bp | Cyanobacteria |  |  |  |
|  | [Synechococcus sp. JA-2-3B'a(2-13)](http://www.ncbi.nlm.nih.gov/Taxonomy/Browser/wwwtax.cgi?id=321332) |  | [NC_007776](http://www.ncbi.nlm.nih.gov/genomes/framik.cgi?db=Genome&gi=19193) | 3046682 bp | Cyanobacteria |  |  |  |
|  | [Synechococcus sp. JA-3-3Ab](http://www.ncbi.nlm.nih.gov/Taxonomy/Browser/wwwtax.cgi?id=321327) |  | [NC_007775](http://www.ncbi.nlm.nih.gov/genomes/framik.cgi?db=Genome&gi=19192) | 2932766 bp | Cyanobacteria |  |  |  |
|  | [Synechococcus sp. WH 8102](http://www.ncbi.nlm.nih.gov/Taxonomy/Browser/wwwtax.cgi?id=84588) |  | [NC_005070](http://www.ncbi.nlm.nih.gov/genomes/framik.cgi?db=Genome&gi=320) | 2434428 bp | Cyanobacteria |  |  |  |
|  | [Synechocystis sp. PCC 6803](http://www.ncbi.nlm.nih.gov/Taxonomy/Browser/wwwtax.cgi?id=1148) |  | [NC_000911](http://www.ncbi.nlm.nih.gov/genomes/framik.cgi?db=Genome&gi=112) | 3573470 bp | Cyanobacteria |  |  |  |
|  | [Syntrophobacter fumaroxidans MPOB](http://www.ncbi.nlm.nih.gov/Taxonomy/Browser/wwwtax.cgi?id=335543) |  | [NC_008554](http://www.ncbi.nlm.nih.gov/entrez/query.fcgi?db=genome&cmd=Retrieve&dopt=Overview&list_uids=20044) | 4990251 bp | Proteobacteria; Deltaproteobacteria |  |  |  |
|  | [Syntrophomonas wolfei subsp. wolfei str. Goettingen](http://www.ncbi.nlm.nih.gov/entrez/query.fcgi?db=genomeprj&cmd=PureSearch&details_term=txid335541%5Borgn%5D AND pt_default%5Bprop%5D ) |  | [NC_008346](http://www.ncbi.nlm.nih.gov/entrez/query.fcgi?db=genome&cmd=Retrieve&dopt=Overview&list_uids=19796) | 2936195 bp | Firmicutes |  |  | S-PM2, KVP40, P-SSM2, P-SSM4 |
|  | [Syntrophus aciditrophicus SB](http://www.ncbi.nlm.nih.gov/Taxonomy/Browser/wwwtax.cgi?id=56780) |  | [NC_007759](http://www.ncbi.nlm.nih.gov/genomes/framik.cgi?db=Genome&gi=19176) | 3179300 bp | Proteobacteria; Deltaproteobacteria |  |  | P2 |
|  | [Thermoanaerobacter tengcongensis MB4](http://www.ncbi.nlm.nih.gov/Taxonomy/Browser/wwwtax.cgi?id=273068) |  | [NC_003869](http://www.ncbi.nlm.nih.gov/genomes/framik.cgi?db=Genome&gi=237) | 2689445 bp | Firmicutes |  |  |  |
|  | [Thermosynechococcus elongatus BP-1](http://www.ncbi.nlm.nih.gov/Taxonomy/Browser/wwwtax.cgi?id=197221) |  | [NC_004113](http://www.ncbi.nlm.nih.gov/genomes/framik.cgi?db=Genome&gi=251) | 2593857 bp | Cyanobacteria |  |  |  |
|  | [Thermotoga maritima MSB8](http://www.ncbi.nlm.nih.gov/Taxonomy/Browser/wwwtax.cgi?id=243274) |  | [NC_000853](http://www.ncbi.nlm.nih.gov/genomes/framik.cgi?db=Genome&gi=141) | 1860725 bp | Thermotogae |  |  |  |
|  | [Thermus thermophilus HB27](http://www.ncbi.nlm.nih.gov/Taxonomy/Browser/wwwtax.cgi?id=262724) |  | [NC_005835](http://www.ncbi.nlm.nih.gov/genomes/framik.cgi?db=Genome&gi=398) | 1894877 bp | Deinococcus-Thermus |  |  |  |
|  | [Thermus thermophilus HB8](http://www.ncbi.nlm.nih.gov/Taxonomy/Browser/wwwtax.cgi?id=300852) |  | [NC_006461](http://www.ncbi.nlm.nih.gov/genomes/framik.cgi?db=Genome&gi=530) | 1849742 bp | Deinococcus-Thermus |  |  |  |
|  | [Thiobacillus denitrificans ATCC 25259](http://www.ncbi.nlm.nih.gov/Taxonomy/Browser/wwwtax.cgi?id=292415) |  | [NC_007404](http://www.ncbi.nlm.nih.gov/genomes/framik.cgi?db=Genome&gi=18750) | 2909809 bp | Deinococcus-Thermus |  |  |  |
|  | [Thiomicrospira crunogena XCL-2](http://www.ncbi.nlm.nih.gov/Taxonomy/Browser/wwwtax.cgi?id=317025) |  | [NC_007520](http://www.ncbi.nlm.nih.gov/genomes/framik.cgi?db=Genome&gi=18916) | 2427734 bp | Proteobacteria; Gammaproteobacteria |  | VHML | VP16C, VHML, λ |
|  | [Thiomicrospira denitrificans ATCC 33889](http://www.ncbi.nlm.nih.gov/Taxonomy/Browser/wwwtax.cgi?id=326298) |  | [NC_007575](http://www.ncbi.nlm.nih.gov/genomes/framik.cgi?db=Genome&gi=18970) | 2201561 bp | Proteobacteria; Epsilonproteobacteria |  |  |  |
|  | [Trichodesmium erythraeum IMS101](http://www.ncbi.nlm.nih.gov/Taxonomy/Browser/wwwtax.cgi?id=203124) |  | [NC_008312](http://www.ncbi.nlm.nih.gov/genomes/framik.cgi?db=Genome&gi=19735) | 7750108 bp | Cyanobacteria |  |  |  |
|  | [Vibrio cholerae O1 biovar eltor str. N16961](http://www.ncbi.nlm.nih.gov/Taxonomy/Browser/wwwtax.cgi?id=243277) | 1 | [NC_002505](http://www.ncbi.nlm.nih.gov/genomes/framik.cgi?db=Genome&gi=161) | 2961149 bp | Proteobacteria; Gammaproteobacteria |  |  |  |
|  | [Vibrio cholerae O1 biovar eltor str. N16961](http://www.ncbi.nlm.nih.gov/Taxonomy/Browser/wwwtax.cgi?id=243277) | 2 | [NC_002506](http://www.ncbi.nlm.nih.gov/genomes/framik.cgi?db=Genome&gi=162) | 1072315 bp | Proteobacteria; Gammaproteobacteria |  |  |  |
|  | [Vibrio fischeri ES114](http://www.ncbi.nlm.nih.gov/Taxonomy/Browser/wwwtax.cgi?id=312309) | 1 | [NC_006840](http://www.ncbi.nlm.nih.gov/genomes/framik.cgi?db=Genome&gi=633) | 2906179 bp | Proteobacteria; Gammaproteobacteria |  | P2 | P2 |
|  | [Vibrio fischeri ES114](http://www.ncbi.nlm.nih.gov/Taxonomy/Browser/wwwtax.cgi?id=312309) | 2 | [NC_006841](http://www.ncbi.nlm.nih.gov/genomes/framik.cgi?db=Genome&gi=634) | 1332022 bp | Proteobacteria; Gammaproteobacteria |  |  |  |
|  | [**Vibrio parahaemolyticus RIMD 2210633**](http://www.ncbi.nlm.nih.gov/Taxonomy/Browser/wwwtax.cgi?id=223926) | **1** | [**NC_004603**](http://www.ncbi.nlm.nih.gov/genomes/framik.cgi?db=Genome&gi=286) | **3288558 bp** | **Proteobacteria; Gammaproteobacteria** | **1667808-1680206** |  |  |
|  | [**Vibrio parahaemolyticus RIMD 2210633**](http://www.ncbi.nlm.nih.gov/Taxonomy/Browser/wwwtax.cgi?id=223926) | **2** | [**NC_004605**](http://www.ncbi.nlm.nih.gov/genomes/framik.cgi?db=Genome&gi=287) | **1877212 bp** | **Proteobacteria; Gammaproteobacteria** | **933864-946277** |  |  |
|  | [Vibrio vulnificus CMCP6](http://www.ncbi.nlm.nih.gov/Taxonomy/Browser/wwwtax.cgi?id=216895) | 1 | [NC_004459](http://www.ncbi.nlm.nih.gov/genomes/framik.cgi?db=Genome&gi=269) | 3281945 bp | Proteobacteria; Gammaproteobacteria |  |  |  |
|  | [**Vibrio vulnificus CMCP6**](http://www.ncbi.nlm.nih.gov/Taxonomy/Browser/wwwtax.cgi?id=216895) | **2** | [**NC_004460**](http://www.ncbi.nlm.nih.gov/genomes/framik.cgi?db=Genome&gi=270) | **1844853 bp** | **Proteobacteria; Gammaproteobacteria** | **314236-325618** |  |  |
|  | [Vibrio vulnificus YJ016](http://www.ncbi.nlm.nih.gov/Taxonomy/Browser/wwwtax.cgi?id=196600) | 1 | [NC_005139](http://www.ncbi.nlm.nih.gov/genomes/framik.cgi?db=Genome&gi=348) | 3354505 bp | Proteobacteria; Gammaproteobacteria |  |  | S-PM2, P-SSM2, P-SSM4 |
|  | [Vibrio vulnificus YJ016](http://www.ncbi.nlm.nih.gov/Taxonomy/Browser/wwwtax.cgi?id=196600) | 2 | [NC_005140](http://www.ncbi.nlm.nih.gov/genomes/framik.cgi?db=Genome&gi=349) | 1857073 bp | Proteobacteria; Gammaproteobacteria |  |  |  |
|  |  |  |  |  |  |  |  |  |
|  | Species with draft assemblies of the genomic sequences |  |  |  |  |  |  |  |
|  | [Acidiphilium cryptum JF-5](http://www.ncbi.nlm.nih.gov/Taxonomy/Browser/wwwtax.cgi?id=349163) |  | [NZ_AAOO00000000](http://www.ncbi.nlm.nih.gov/genomes/framik.cgi?db=Genome&gi=5444) | 3917149 bp | Proteobacteria; Alphaproteobacteria |  |  |  |
|  | [Algoriphagus sp. PR1](http://www.ncbi.nlm.nih.gov/Taxonomy/Browser/wwwtax.cgi?id=388413) |  | [NZ_AAXU00000000](http://www.ncbi.nlm.nih.gov/genomes/framik.cgi?db=Genome&gi=5626) | 4776234 bp | Bacteroidetes/Chlorobi group |  |  |  |
|  | [Alkaliphilus metalliredigenes QYMF](http://www.ncbi.nlm.nih.gov/Taxonomy/Browser/wwwtax.cgi?id=293826) |  | [NZ_AAKU00000000](http://www.ncbi.nlm.nih.gov/genomes/framik.cgi?db=Genome&gi=5360) | 4410303 bp | Firmicutes |  |  | S-PM2, KVP40, P-SSM2, P-SSM4 |
|  | [Alpha proteobacterium HTCC2255](http://www.ncbi.nlm.nih.gov/Taxonomy/Browser/wwwtax.cgi?id=373154) |  | [NZ_AATR00000000](http://www.ncbi.nlm.nih.gov/genomes/framik.cgi?db=Genome&gi=5521) | 4812704 bp | Proteobacteria; Alphaproteobacteria |  |  |  |
|  | [Alteromonadales bacterium TW-7](http://www.ncbi.nlm.nih.gov/Taxonomy/Browser/wwwtax.cgi?id=156578) |  | [NZ_AAVS00000000](http://www.ncbi.nlm.nih.gov/genomes/framik.cgi?db=Genome&gi=5583) | 4104952 bp | Proteobacteria; Gammaproteobacteria |  |  |  |
|  | [Alteromonas macleodii 'Deep ecotype'](http://www.ncbi.nlm.nih.gov/Taxonomy/Browser/wwwtax.cgi?id=314275) |  | [NZ_AAOD00000000](http://www.ncbi.nlm.nih.gov/genomes/framik.cgi?db=Genome&gi=5433) | 4413342 bp | Proteobacteria; Gammaproteobacteria |  |  | VpV262 |
|  | [Aurantimonas sp. SI85-9A1](http://www.ncbi.nlm.nih.gov/Taxonomy/Browser/wwwtax.cgi?id=314269) |  | [NZ_AAPJ00000000](http://www.ncbi.nlm.nih.gov/genomes/framik.cgi?db=Genome&gi=5464) | 4325257 bp | Proteobacteria; Alphaproteobacteria |  |  |  |
|  | [Bacillus sp. B14905](http://www.ncbi.nlm.nih.gov/Taxonomy/Browser/wwwtax.cgi?id=388400) |  | [NZ_AAXV00000000](http://www.ncbi.nlm.nih.gov/genomes/framik.cgi?db=Genome&gi=5627) | 4497271 bp | Firmicutes |  |  | φHSIC |
|  | [Bacillus sp. NRRL B-14911](http://www.ncbi.nlm.nih.gov/Taxonomy/Browser/wwwtax.cgi?id=313627) |  | [NZ_AAOX00000000](http://www.ncbi.nlm.nih.gov/genomes/framik.cgi?db=Genome&gi=5452) | 5085825 bp | Firmicutes |  |  |  |
|  | [Blastopirellula marina DSM 3645](http://www.ncbi.nlm.nih.gov/Taxonomy/Browser/wwwtax.cgi?id=314230) |  | [NZ_AANZ00000000](http://www.ncbi.nlm.nih.gov/genomes/framik.cgi?db=Genome&gi=5429) | 6653746 bp | Planctomycetes |  |  |  |
|  | [Caldicellulosiruptor saccharolyticus DSM 8903](http://www.ncbi.nlm.nih.gov/Taxonomy/Browser/wwwtax.cgi?id=351627) |  | [NZ_AALW00000000](http://www.ncbi.nlm.nih.gov/genomes/framik.cgi?db=Genome&gi=5379) | 2788317 bp | Firmicutes |  |  |  |
|  | [Candidatus Desulfococcus oleovorans Hxd3](http://www.ncbi.nlm.nih.gov/Taxonomy/Browser/wwwtax.cgi?id=96561) |  | [NZ_AAWN00000000](http://www.ncbi.nlm.nih.gov/genomes/framik.cgi?db=Genome&gi=5606) | 3788421 bp | Proteobacteria; Deltaproteobacteria |  |  |  |
|  | [Candidatus Pelagibacter ubique HTCC1002](http://www.ncbi.nlm.nih.gov/Taxonomy/Browser/wwwtax.cgi?id=314261) |  | [NZ_AAPV00000000](http://www.ncbi.nlm.nih.gov/genomes/framik.cgi?db=Genome&gi=5471) | 1327604 bp | Proteobacteria; Alphaproteobacteria |  |  |  |
|  | [Cellulophaga sp. MED134](http://www.ncbi.nlm.nih.gov/Taxonomy/Browser/wwwtax.cgi?id=313590) |  | [NZ_AAMZ00000000](http://www.ncbi.nlm.nih.gov/genomes/framik.cgi?db=Genome&gi=5415) | 3301953 bp | Bacteroidetes/Chlorobi group |  |  |  |
|  | [Chlorobium ferrooxidans DSM 13031](http://www.ncbi.nlm.nih.gov/Taxonomy/Browser/wwwtax.cgi?id=377431) |  | [NZ_AASE00000000](http://www.ncbi.nlm.nih.gov/genomes/framik.cgi?db=Genome&gi=5503) | 2538957 bp | Bacteroidetes/Chlorobi group |  |  |  |
|  | [Chlorobium limicola DSM 245](http://www.ncbi.nlm.nih.gov/Taxonomy/Browser/wwwtax.cgi?id=290315) |  | [NZ_AAHJ00000000](http://www.ncbi.nlm.nih.gov/genomes/framik.cgi?db=Genome&gi=5303) | 2747025 bp | Bacteroidetes/Chlorobi group |  |  |  |
|  | [Chlorobium phaeobacteroides BS1](http://www.ncbi.nlm.nih.gov/Taxonomy/Browser/wwwtax.cgi?id=331678) |  | [NZ_AAIC00000000](http://www.ncbi.nlm.nih.gov/genomes/framik.cgi?db=Genome&gi=5306) | 4444192 bp | Bacteroidetes/Chlorobi group |  |  |  |
|  | [Chloroflexus aggregans DSM 9485](http://www.ncbi.nlm.nih.gov/Taxonomy/Browser/wwwtax.cgi?id=326427) |  | [NZ_AAUI00000000](http://www.ncbi.nlm.nih.gov/genomes/framik.cgi?db=Genome&gi=5551) | 4596772 bp | Chloroflexi |  |  |  |
|  | [Chloroflexus aurantiacus J-10-fl](http://www.ncbi.nlm.nih.gov/Taxonomy/Browser/wwwtax.cgi?id=324602) |  | [NZ_AAAH00000000](http://www.ncbi.nlm.nih.gov/genomes/framik.cgi?db=Genome&gi=5018) | 5193782 bp | Chloroflexi |  |  |  |
|  | [Clostridium sp. OhILAs](http://www.ncbi.nlm.nih.gov/Taxonomy/Browser/wwwtax.cgi?id=350688) |  | [NZ_AAQV00000000](http://www.ncbi.nlm.nih.gov/genomes/framik.cgi?db=Genome&gi=5494) | 2997608 bp | Firmicutes |  |  |  |
|  | [Comamonas testosteroni KF-1](http://www.ncbi.nlm.nih.gov/Taxonomy/Browser/wwwtax.cgi?id=399795) |  | [NZ_AAUJ00000000](http://www.ncbi.nlm.nih.gov/genomes/framik.cgi?db=Genome&gi=5552) | 5906374 bp | Proteobacteria; Betaproteobacteria |  | P2 | P2, φHSIC |
|  | [Congregibacter litoralis KT71](http://www.ncbi.nlm.nih.gov/entrez/query.fcgi?db=genomeprj&cmd=PureSearch&details_term=txid314285%5Borgn%5D AND pt_default%5Bprop%5D ) |  | [NZ_AAOA00000000](http://www.ncbi.nlm.nih.gov/genomes/framik.cgi?db=Genome&gi=5431) | 4328100 bp | Proteobacteria; Gammaproteobacteria |  |  |  |
|  | [Croceibacter atlanticus HTCC2559](http://www.ncbi.nlm.nih.gov/Taxonomy/Browser/wwwtax.cgi?id=216432) |  | [NZ_AAMP00000000](http://www.ncbi.nlm.nih.gov/genomes/framik.cgi?db=Genome&gi=5392) | 2954711 bp | Bacteroidetes/Chlorobi group |  |  |  |
|  | [Crocosphaera watsonii WH 8501](http://www.ncbi.nlm.nih.gov/Taxonomy/Browser/wwwtax.cgi?id=165597) |  | [NZ_AADV00000000](http://www.ncbi.nlm.nih.gov/genomes/framik.cgi?db=Genome&gi=5222) | 6238156 bp | Cyanobacteria |  |  |  |
|  | [Cyanothece sp. CCY0110](http://www.ncbi.nlm.nih.gov/Taxonomy/Browser/wwwtax.cgi?id=391612) |  | [NZ_AAXW00000000](http://www.ncbi.nlm.nih.gov/genomes/framik.cgi?db=Genome&gi=5628) | 5880532 bp | Cyanobacteria |  |  |  |
|  | [Delta proteobacterium MLMS-1](http://www.ncbi.nlm.nih.gov/Taxonomy/Browser/wwwtax.cgi?id=262489) |  | [NZ_AAQF00000000](http://www.ncbi.nlm.nih.gov/genomes/framik.cgi?db=Genome&gi=5477) | 6058912 bp | Proteobacteria; Deltaproteobacteria |  |  |  |
|  | [Desulfitobacterium hafniense DCB-2](http://www.ncbi.nlm.nih.gov/Taxonomy/Browser/wwwtax.cgi?id=272564) |  | [NZ_AAAW00000000](http://www.ncbi.nlm.nih.gov/genomes/framik.cgi?db=Genome&gi=5017) | 5276979 bp | Firmicutes |  |  |  |
|  | [Desulfuromonas acetoxidans DSM 684](http://www.ncbi.nlm.nih.gov/Taxonomy/Browser/wwwtax.cgi?id=281689) |  | [NZ_AAEW00000000](http://www.ncbi.nlm.nih.gov/genomes/framik.cgi?db=Genome&gi=5309) | 3828328 bp | Proteobacteria; Deltaproteobacteria |  |  | VpV262 |
|  | [Dinoroseobacter shibae DFL 12](http://www.ncbi.nlm.nih.gov/Taxonomy/Browser/wwwtax.cgi?id=398580) |  | [NZ_AAVE00000000](http://www.ncbi.nlm.nih.gov/genomes/framik.cgi?db=Genome&gi=5574) | 4327529 bp | Proteobacteria; Alphaproteobacteria |  |  | S-PM2, KVP40 |
|  | [Erythrobacter sp. NAP1](http://www.ncbi.nlm.nih.gov/Taxonomy/Browser/wwwtax.cgi?id=237727) |  | [NZ_AAMW00000000](http://www.ncbi.nlm.nih.gov/genomes/framik.cgi?db=Genome&gi=5412) | 3265346 bp | Proteobacteria; Alphaproteobacteria |  |  |  |
|  | [Exiguobacterium sibiricum 255-15](http://www.ncbi.nlm.nih.gov/Taxonomy/Browser/wwwtax.cgi?id=262543) |  | [NZ_AADW00000000](http://www.ncbi.nlm.nih.gov/genomes/framik.cgi?db=Genome&gi=5224) | 3030158 bp | Firmicutes |  |  |  |
|  | [Fervidobacterium nodosum Rt17-B1](http://www.ncbi.nlm.nih.gov/Taxonomy/Browser/wwwtax.cgi?id=381764) |  | [NZ_AAUK00000000](http://www.ncbi.nlm.nih.gov/genomes/framik.cgi?db=Genome&gi=5553) | 1849124 bp | Thermotogae |  |  |  |
|  | [Flavobacteria bacterium BAL38](http://www.ncbi.nlm.nih.gov/Taxonomy/Browser/wwwtax.cgi?id=391598) |  | [NZ_AAXX00000000](http://www.ncbi.nlm.nih.gov/genomes/framik.cgi?db=Genome&gi=5629) | 2806989 bp | Bacteroidetes/Chlorobi group |  |  |  |
|  | [Flavobacteria bacterium BBFL7](http://www.ncbi.nlm.nih.gov/Taxonomy/Browser/wwwtax.cgi?id=156586) |  | [NZ_AAPD00000000](http://www.ncbi.nlm.nih.gov/genomes/framik.cgi?db=Genome&gi=5459) | 3083153 bp | Bacteroidetes/Chlorobi group |  |  |  |
|  | [Flavobacteriales bacterium HTCC2170](http://www.ncbi.nlm.nih.gov/Taxonomy/Browser/wwwtax.cgi?id=313603) |  | [NZ_AAOC00000000](http://www.ncbi.nlm.nih.gov/genomes/framik.cgi?db=Genome&gi=5432) | 3877161 bp | Bacteroidetes/Chlorobi group |  |  |  |
|  | [Flavobacterium johnsoniae UW101](http://www.ncbi.nlm.nih.gov/Taxonomy/Browser/wwwtax.cgi?id=376686) |  | [NZ_AAPM00000000](http://www.ncbi.nlm.nih.gov/genomes/framik.cgi?db=Genome&gi=5468) | 6069808 bp | Bacteroidetes/Chlorobi group |  |  |  |
|  | [Fulvimarina pelagi HTCC2506](http://www.ncbi.nlm.nih.gov/Taxonomy/Browser/wwwtax.cgi?id=314231) |  | [NZ_AATP00000000](http://www.ncbi.nlm.nih.gov/genomes/framik.cgi?db=Genome&gi=5519) | 3802689 bp | Proteobacteria; Alphaproteobacteria |  |  |  |
|  | [Geobacter lovleyi SZ](http://www.ncbi.nlm.nih.gov/Taxonomy/Browser/wwwtax.cgi?id=398767) |  | [NZ_AAVG00000000](http://www.ncbi.nlm.nih.gov/genomes/framik.cgi?db=Genome&gi=5576) | 3871860 bp | Proteobacteria; Deltaproteobacteria |  |  |  |
|  | [Geobacter sp. FRC-32](http://www.ncbi.nlm.nih.gov/Taxonomy/Browser/wwwtax.cgi?id=316067) |  | [NZ_AASH00000000](http://www.ncbi.nlm.nih.gov/genomes/framik.cgi?db=Genome&gi=5504) | 3982463 bp | Proteobacteria; Deltaproteobacteria |  |  |  |
|  | [Geobacter uraniumreducens Rf4](http://www.ncbi.nlm.nih.gov/Taxonomy/Browser/wwwtax.cgi?id=351605) |  | [NZ_AAON00000000](http://www.ncbi.nlm.nih.gov/genomes/framik.cgi?db=Genome&gi=5443) | 4882254 bp | Proteobacteria; Deltaproteobacteria |  |  |  |
|  | [Halothermothrix orenii H 168](http://www.ncbi.nlm.nih.gov/Taxonomy/Browser/wwwtax.cgi?id=373903) |  | [NZ_AAOZ00000000](http://www.ncbi.nlm.nih.gov/genomes/framik.cgi?db=Genome&gi=5456) | 2463968 bp | Firmicutes |  |  |  |
|  | [Idiomarina baltica OS145](http://www.ncbi.nlm.nih.gov/Taxonomy/Browser/wwwtax.cgi?id=314276) |  | [NZ_AAMX00000000](http://www.ncbi.nlm.nih.gov/genomes/framik.cgi?db=Genome&gi=5413) | 2722127 bp | Proteobacteria; Gammaproteobacteria |  |  |  |
|  | [Janibacter sp. HTCC2649](http://www.ncbi.nlm.nih.gov/Taxonomy/Browser/wwwtax.cgi?id=313589) |  | [NZ_AAMN00000000](http://www.ncbi.nlm.nih.gov/genomes/framik.cgi?db=Genome&gi=5402) | 4228723 bp | Actinobacteria |  |  |  |
|  | [Kineococcus radiotolerans SRS30216](http://www.ncbi.nlm.nih.gov/Taxonomy/Browser/wwwtax.cgi?id=266940) |  | [NZ_AAEF00000000](http://www.ncbi.nlm.nih.gov/genomes/framik.cgi?db=Genome&gi=5228) | 4893957 bp | Actinobacteria |  |  |  |
|  | [Leeuwenhoekiella blandensis MED217](http://www.ncbi.nlm.nih.gov/entrez/query.fcgi?db=genomeprj&cmd=PureSearch&details_term=txid398720%5Borgn%5D AND pt_default%5Bprop%5D ) |  | [NZ_AANC00000000](http://www.ncbi.nlm.nih.gov/genomes/framik.cgi?db=Genome&gi=5418) | 4238065 bp | Bacteroidetes/Chlorobi group |  |  |  |
|  | [Loktanella vestfoldensis SKA53](http://www.ncbi.nlm.nih.gov/Taxonomy/Browser/wwwtax.cgi?id=314232) |  | [NZ_AAMS00000000](http://www.ncbi.nlm.nih.gov/genomes/framik.cgi?db=Genome&gi=5404) | 3063691 bp | Proteobacteria; Alphaproteobacteria |  |  |  |
|  | [Lyngbya sp. PCC 8106](http://www.ncbi.nlm.nih.gov/Taxonomy/Browser/wwwtax.cgi?id=313612) |  | [NZ_AAVU00000000](http://www.ncbi.nlm.nih.gov/genomes/framik.cgi?db=Genome&gi=5585) | 7037511 bp | Cyanobacteria |  |  |  |
|  | [Magnetospirillum magnetotacticum MS-1](http://www.ncbi.nlm.nih.gov/Taxonomy/Browser/wwwtax.cgi?id=188) |  | [NZ_AAAP00000000](http://www.ncbi.nlm.nih.gov/genomes/framik.cgi?db=Genome&gi=5016) | 9212200 bp | Proteobacteria; Alphaproteobacteria |  | VHML, φHSIC | VP16C, VHML, λ |
|  | [Marine actinobacterium PHSC20C1](http://www.ncbi.nlm.nih.gov/Taxonomy/Browser/wwwtax.cgi?id=312284) |  | [NZ_AAOB00000000](http://www.ncbi.nlm.nih.gov/genomes/framik.cgi?db=Genome&gi=5439) | 2769012 bp | Actinobacteria |  |  |  |
|  | [Marine gamma proteobacterium HTCC2080](http://www.ncbi.nlm.nih.gov/Taxonomy/Browser/wwwtax.cgi?id=247639) |  | [NZ_AAVV00000000](http://www.ncbi.nlm.nih.gov/genomes/framik.cgi?db=Genome&gi=5587) | 3576081 bp | Proteobacteria; Gammaproteobacteria |  |  |  |
|  | [Marine gamma proteobacterium HTCC2143](http://www.ncbi.nlm.nih.gov/Taxonomy/Browser/wwwtax.cgi?id=247633) |  | [NZ_AAVT00000000](http://www.ncbi.nlm.nih.gov/genomes/framik.cgi?db=Genome&gi=5584) | 3925629 bp | Proteobacteria; Gammaproteobacteria |  |  |  |
|  | [Marine gamma proteobacterium HTCC2207](http://www.ncbi.nlm.nih.gov/Taxonomy/Browser/wwwtax.cgi?id=247635) |  | [NZ_AAPI00000000](http://www.ncbi.nlm.nih.gov/genomes/framik.cgi?db=Genome&gi=5463) | 2620870 bp | Proteobacteria; Gammaproteobacteria |  |  |  |
|  | [Marinobacter sp. ELB17](http://www.ncbi.nlm.nih.gov/Taxonomy/Browser/wwwtax.cgi?id=270374) |  | [NZ_AAXY00000000](http://www.ncbi.nlm.nih.gov/genomes/framik.cgi?db=Genome&gi=5630) | 4894744 bp | Proteobacteria; Gammaproteobacteria |  |  |  |
|  | [Marinomonas sp. MED121](http://www.ncbi.nlm.nih.gov/Taxonomy/Browser/wwwtax.cgi?id=314277) |  | [NZ_AANE00000000](http://www.ncbi.nlm.nih.gov/genomes/framik.cgi?db=Genome&gi=5424) | 5121076 bp | Proteobacteria; Gammaproteobacteria |  |  |  |
|  | [Marinomonas sp. MWYL1](http://www.ncbi.nlm.nih.gov/Taxonomy/Browser/wwwtax.cgi?id=400668) |  | [NZ_AAVH00000000](http://www.ncbi.nlm.nih.gov/genomes/framik.cgi?db=Genome&gi=5577) | 5039593 bp | Proteobacteria; Gammaproteobacteria |  | P2 | P2 |
|  | [Mariprofundus ferrooxydans PV-1](http://www.ncbi.nlm.nih.gov/Taxonomy/Browser/wwwtax.cgi?id=314345) |  | [NZ_AATS00000000](http://www.ncbi.nlm.nih.gov/genomes/framik.cgi?db=Genome&gi=5522) | 2867087 bp | Proteobacteria; unclassified Proteobacteria |  |  |  |
|  | [Methylophilales bacterium HTCC2181](http://www.ncbi.nlm.nih.gov/Taxonomy/Browser/wwwtax.cgi?id=383631) |  | [NZ_AAUX00000000](http://www.ncbi.nlm.nih.gov/genomes/framik.cgi?db=Genome&gi=5567) | 1304428 bp | Proteobacteria; Betaproteobacteria |  |  |  |
|  | [Microscilla marina ATCC 23134](http://www.ncbi.nlm.nih.gov/Taxonomy/Browser/wwwtax.cgi?id=313606) |  | [NZ_AAWS00000000](http://www.ncbi.nlm.nih.gov/genomes/framik.cgi?db=Genome&gi=5612) | 9771226 bp | Bacteroidetes/Chlorobi group |  |  |  |
|  | [Nitrobacter sp. Nb-311A](http://www.ncbi.nlm.nih.gov/Taxonomy/Browser/wwwtax.cgi?id=314253) |  | [NZ_AAMY00000000](http://www.ncbi.nlm.nih.gov/genomes/framik.cgi?db=Genome&gi=5414) | 4104352 bp | Proteobacteria; Alphaproteobacteria |  |  | VP16C, S-PM2, VHML, P-SSM2, P-SSM4, λ |
|  | [Nitrococcus mobilis Nb-231](http://www.ncbi.nlm.nih.gov/Taxonomy/Browser/wwwtax.cgi?id=314278) |  | [NZ_AAOF00000000](http://www.ncbi.nlm.nih.gov/genomes/framik.cgi?db=Genome&gi=5438) | 3617638 bp | Proteobacteria; Gammaproteobacteria |  |  |  |
|  | [Nodularia spumigena CCY9414](http://www.ncbi.nlm.nih.gov/Taxonomy/Browser/wwwtax.cgi?id=313624) |  | [NZ_AAVW00000000](http://www.ncbi.nlm.nih.gov/genomes/framik.cgi?db=Genome&gi=5588) | 5316258 bp | Cyanobacteria |  |  |  |
|  | [Nostoc punctiforme](http://www.ncbi.nlm.nih.gov/Taxonomy/Browser/wwwtax.cgi?id=63737) |  | [NZ_AAAY00000000](http://www.ncbi.nlm.nih.gov/genomes/framik.cgi?db=Genome&gi=5020) | 9020037 bp | Cyanobacteria |  |  |  |
|  | [Oceanicaulis alexandrii HTCC2633](http://www.ncbi.nlm.nih.gov/Taxonomy/Browser/wwwtax.cgi?id=314254) |  | [NZ_AAMQ00000000](http://www.ncbi.nlm.nih.gov/genomes/framik.cgi?db=Genome&gi=5394) | 3168201 bp | Proteobacteria; Alphaproteobacteria |  |  | VP16C, VHML, λ |
|  | [Oceanicola batsensis HTCC2597](http://www.ncbi.nlm.nih.gov/Taxonomy/Browser/wwwtax.cgi?id=252305) |  | [NZ_AAMO00000000](http://www.ncbi.nlm.nih.gov/genomes/framik.cgi?db=Genome&gi=5403) | 4437668 bp | Proteobacteria; Alphaproteobacteria |  |  |  |
|  | [Oceanicola granulosus HTCC2516](http://www.ncbi.nlm.nih.gov/Taxonomy/Browser/wwwtax.cgi?id=314256) |  | [NZ_AAOT00000000](http://www.ncbi.nlm.nih.gov/genomes/framik.cgi?db=Genome&gi=5448) | 4039111 bp | Proteobacteria; Alphaproteobacteria |  |  |  |
|  | [**Oceanobacter sp. RED65**](http://www.ncbi.nlm.nih.gov/Taxonomy/Browser/wwwtax.cgi?id=207949) |  | [**NZ_AAQH00000000**](http://www.ncbi.nlm.nih.gov/genomes/framik.cgi?db=Genome&gi=5482) | **3527084 bp** | **Proteobacteria; Gammaproteobacteria** | **311869-322167 (AAQH01000002)** |  |  |
|  | [Oceanospirillum sp. MED92](http://www.ncbi.nlm.nih.gov/Taxonomy/Browser/wwwtax.cgi?id=207954) |  | [NZ_AAOW00000000](http://www.ncbi.nlm.nih.gov/genomes/framik.cgi?db=Genome&gi=5451) | 3873219 bp | Proteobacteria; Gammaproteobacteria |  |  |  |
|  | [Oenococcus oeni ATCC BAA-1163](http://www.ncbi.nlm.nih.gov/Taxonomy/Browser/wwwtax.cgi?id=379360) |  | [NZ_AAUV00000000](http://www.ncbi.nlm.nih.gov/genomes/framik.cgi?db=Genome&gi=5565) | 1753447 bp | Firmicutes |  |  |  |
|  | [Parvibaculum lavamentivorans DS-1](http://www.ncbi.nlm.nih.gov/Taxonomy/Browser/wwwtax.cgi?id=402881) |  | [NZ_AAWJ00000000](http://www.ncbi.nlm.nih.gov/genomes/framik.cgi?db=Genome&gi=5602) | 3854587 bp | Proteobacteria; Alphaproteobacteria |  |  |  |
|  | [Parvularcula bermudensis HTCC2503](http://www.ncbi.nlm.nih.gov/Taxonomy/Browser/wwwtax.cgi?id=314260) |  | [NZ_AAMU00000000](http://www.ncbi.nlm.nih.gov/genomes/framik.cgi?db=Genome&gi=5407) | 2907267 bp | Proteobacteria; Alphaproteobacteria |  |  |  |
|  | [Pelodictyon phaeoclathratiforme BU-1](http://www.ncbi.nlm.nih.gov/Taxonomy/Browser/wwwtax.cgi?id=324925) |  | [NZ_AAIK00000000](http://www.ncbi.nlm.nih.gov/genomes/framik.cgi?db=Genome&gi=5315) | 2967835 bp | Bacteroidetes/Chlorobi group |  |  |  |
|  | [Petrotoga mobilis SJ95](http://www.ncbi.nlm.nih.gov/Taxonomy/Browser/wwwtax.cgi?id=403833) |  | [NZ_AAZB00000000](http://www.ncbi.nlm.nih.gov/genomes/framik.cgi?db=Genome&gi=5657) | 2139960 bp | Thermotogae |  |  |  |
|  | [**Photobacterium profundum 3TCK**](http://www.ncbi.nlm.nih.gov/Taxonomy/Browser/wwwtax.cgi?id=314280) |  | [**NZ_AAPH00000000**](http://www.ncbi.nlm.nih.gov/genomes/framik.cgi?db=Genome&gi=5462) | **6107610 bp** | **Proteobacteria; Gammaproteobacteria** | **10536-24524 (AAPH01000002)** |  |  |
|  | [Photobacterium sp. SKA34](http://www.ncbi.nlm.nih.gov/Taxonomy/Browser/wwwtax.cgi?id=121723) |  | [NZ_AAOU00000000](http://www.ncbi.nlm.nih.gov/genomes/framik.cgi?db=Genome&gi=5449) | 4946988 bp | Proteobacteria; Gammaproteobacteria |  |  |  |
|  | [Polaribacter irgensii 23-P](http://www.ncbi.nlm.nih.gov/Taxonomy/Browser/wwwtax.cgi?id=313594) |  | [NZ_AAOG00000000](http://www.ncbi.nlm.nih.gov/genomes/framik.cgi?db=Genome&gi=5435) | 2745458 bp | Bacteroidetes/Chlorobi group |  |  |  |
|  | [Prochlorococcus marinus str. MIT 9211](http://www.ncbi.nlm.nih.gov/Taxonomy/Browser/wwwtax.cgi?id=93059) |  | [NZ_AALP00000000](http://www.ncbi.nlm.nih.gov/genomes/framik.cgi?db=Genome&gi=5405) | 1839003 bp | Cyanobacteria |  |  |  |
|  | [Prosthecochloris aestuarii DSM 271](http://www.ncbi.nlm.nih.gov/Taxonomy/Browser/wwwtax.cgi?id=290512) |  | [NZ_AAIJ00000000](http://www.ncbi.nlm.nih.gov/genomes/framik.cgi?db=Genome&gi=5316) | 2540059 bp | Bacteroidetes/Chlorobi group |  |  |  |
|  | [Pseudoalteromonas tunicata D2](http://www.ncbi.nlm.nih.gov/Taxonomy/Browser/wwwtax.cgi?id=87626) |  | [NZ_AAOH00000000](http://www.ncbi.nlm.nih.gov/genomes/framik.cgi?db=Genome&gi=5440) | 4982425 bp | Proteobacteria; Gammaproteobacteria |  | P2 | P2 |
|  | [Pseudomonas putida F1](http://www.ncbi.nlm.nih.gov/Taxonomy/Browser/wwwtax.cgi?id=351746) |  | [NZ_AALM00000000](http://www.ncbi.nlm.nih.gov/genomes/framik.cgi?db=Genome&gi=5382) | 5925059 bp | Proteobacteria; Gammaproteobacteria |  | φHSIC |  |
|  | [Psychroflexus torquis ATCC 700755](http://www.ncbi.nlm.nih.gov/Taxonomy/Browser/wwwtax.cgi?id=313595) |  | [NZ_AAPR00000000](http://www.ncbi.nlm.nih.gov/genomes/framik.cgi?db=Genome&gi=5469) | 6014448 bp | Bacteroidetes/Chlorobi group |  | T7, VP4, P-SSP7 | S-PM2, φJL001 |
|  | [Psychromonas sp. CNPT3](http://www.ncbi.nlm.nih.gov/Taxonomy/Browser/wwwtax.cgi?id=314282) |  | [NZ_AAPG00000000](http://www.ncbi.nlm.nih.gov/genomes/framik.cgi?db=Genome&gi=5461) | 2945265 bp | Proteobacteria; Gammaproteobacteria |  |  |  |
|  | [Ralstonia pickettii 12J](http://www.ncbi.nlm.nih.gov/Taxonomy/Browser/wwwtax.cgi?id=402626) |  | [NZ_AAWK00000000](http://www.ncbi.nlm.nih.gov/genomes/framik.cgi?db=Genome&gi=5603) | 5260531 bp | Proteobacteria; Betaproteobacteria |  |  | VpV262 |
|  | [Reinekea sp. MED297](http://www.ncbi.nlm.nih.gov/Taxonomy/Browser/wwwtax.cgi?id=314283) |  | [NZ_AAOE00000000](http://www.ncbi.nlm.nih.gov/genomes/framik.cgi?db=Genome&gi=5434) | 4506331 bp | Proteobacteria; Gammaproteobacteria |  |  |  |
|  | [Rhodobacterales bacterium HTCC2150](http://www.ncbi.nlm.nih.gov/Taxonomy/Browser/wwwtax.cgi?id=388401) |  | [NZ_AAXZ00000000](http://www.ncbi.nlm.nih.gov/genomes/framik.cgi?db=Genome&gi=5631) | 3582902 bp | Proteobacteria; Alphaproteobacteria |  |  |  |
|  | [Rhodobacterales bacterium HTCC2654](http://www.ncbi.nlm.nih.gov/Taxonomy/Browser/wwwtax.cgi?id=314271) |  | [NZ_AAMT00000000](http://www.ncbi.nlm.nih.gov/genomes/framik.cgi?db=Genome&gi=5406) | 4529231 bp | Proteobacteria; Alphaproteobacteria |  | VHML | VP16C, VHML |
|  | [Robiginitalea biformata HTCC2501](http://www.ncbi.nlm.nih.gov/Taxonomy/Browser/wwwtax.cgi?id=313596) |  | [NZ_AAOI00000000](http://www.ncbi.nlm.nih.gov/genomes/framik.cgi?db=Genome&gi=5436) | 3534942 bp | Bacteroidetes/Chlorobi group |  |  |  |
|  | [Roseiflexus castenholzii DSM 13941](http://www.ncbi.nlm.nih.gov/Taxonomy/Browser/wwwtax.cgi?id=383372) |  | [NZ_AAUM00000000](http://www.ncbi.nlm.nih.gov/genomes/framik.cgi?db=Genome&gi=5555) | 5691558 bp | Chloroflexi |  |  |  |
|  | [Roseiflexus sp. RS-1](http://www.ncbi.nlm.nih.gov/Taxonomy/Browser/wwwtax.cgi?id=357808) |  | [NZ_AAQU00000000](http://www.ncbi.nlm.nih.gov/genomes/framik.cgi?db=Genome&gi=5493) | 5827770 bp | Chloroflexi |  |  |  |
|  | [Roseobacter sp. CCS2](http://www.ncbi.nlm.nih.gov/Taxonomy/Browser/wwwtax.cgi?id=391593) |  | [NZ_AAYB00000000](http://www.ncbi.nlm.nih.gov/genomes/framik.cgi?db=Genome&gi=5633) | 3497325 bp | Proteobacteria; Alphaproteobacteria |  |  |  |
|  | [Roseobacter sp. MED193](http://www.ncbi.nlm.nih.gov/Taxonomy/Browser/wwwtax.cgi?id=314262) |  | [NZ_AANB00000000](http://www.ncbi.nlm.nih.gov/genomes/framik.cgi?db=Genome&gi=5417) | 4652716 bp | Proteobacteria; Alphaproteobacteria |  | VHML | VP16C, VHML, λ |
|  | [Roseobacter sp. SK209-2-6](http://www.ncbi.nlm.nih.gov/Taxonomy/Browser/wwwtax.cgi?id=388739) |  | [NZ_AAYC00000000](http://www.ncbi.nlm.nih.gov/genomes/framik.cgi?db=Genome&gi=5634) | 4555826 bp | Proteobacteria; Alphaproteobacteria |  |  |  |
|  | [Roseovarius nubinhibens ISM](http://www.ncbi.nlm.nih.gov/Taxonomy/Browser/wwwtax.cgi?id=89187) |  | [NZ_AALY00000000](http://www.ncbi.nlm.nih.gov/genomes/framik.cgi?db=Genome&gi=5395) | 3668667 bp | Proteobacteria; Alphaproteobacteria |  |  |  |
|  | [Roseovarius sp. 217](http://www.ncbi.nlm.nih.gov/Taxonomy/Browser/wwwtax.cgi?id=314264) |  | [NZ_AAMV00000000](http://www.ncbi.nlm.nih.gov/genomes/framik.cgi?db=Genome&gi=5411) | 4762632 bp | Proteobacteria; Alphaproteobacteria |  |  |  |
|  | [Roseovarius sp. HTCC2601](http://www.ncbi.nlm.nih.gov/Taxonomy/Browser/wwwtax.cgi?id=314265) |  | [NZ_AATQ00000000](http://www.ncbi.nlm.nih.gov/genomes/framik.cgi?db=Genome&gi=5520) | 5425920 bp | Proteobacteria; Alphaproteobacteria |  | VHML | VP16C, VHML, λ |
|  | [Sagittula stellata E-37](http://www.ncbi.nlm.nih.gov/Taxonomy/Browser/wwwtax.cgi?id=388399) |  | [NZ_AAYA00000000](http://www.ncbi.nlm.nih.gov/genomes/framik.cgi?db=Genome&gi=5632) | 5262893 bp | Proteobacteria; Alphaproteobacteria |  |  | VP16C, VHML, λ |
|  | [Salinispora arenicola CNS205](http://www.ncbi.nlm.nih.gov/Taxonomy/Browser/wwwtax.cgi?id=391037) |  | [NZ_AAWA00000000](http://www.ncbi.nlm.nih.gov/genomes/framik.cgi?db=Genome&gi=5592) | 5733399 bp | Actinobacteria |  |  |  |
|  | [Shewanella baltica OS185](http://www.ncbi.nlm.nih.gov/Taxonomy/Browser/wwwtax.cgi?id=402882) |  | [NZ_AAZC00000000](http://www.ncbi.nlm.nih.gov/genomes/framik.cgi?db=Genome&gi=5658) | 5135142 bp | Proteobacteria; Gammaproteobacteria |  |  | VP16C, VHML, λ |
|  | [Shewanella baltica OS195](http://www.ncbi.nlm.nih.gov/Taxonomy/Browser/wwwtax.cgi?id=399599) |  | [NZ_AATK00000000](http://www.ncbi.nlm.nih.gov/genomes/framik.cgi?db=Genome&gi=5517) | 5310173 bp | Proteobacteria; Gammaproteobacteria |  | P2 | VP16C, P2, VHML, λ |
|  | [Shewanella pealeana ATCC 700345](http://www.ncbi.nlm.nih.gov/Taxonomy/Browser/wwwtax.cgi?id=398579) |  | [NZ_AAVJ00000000](http://www.ncbi.nlm.nih.gov/genomes/framik.cgi?db=Genome&gi=5579) | 4988732 bp | Proteobacteria; Gammaproteobacteria |  |  |  |
|  | [Shewanella putrefaciens 200](http://www.ncbi.nlm.nih.gov/Taxonomy/Browser/wwwtax.cgi?id=399804) |  | [NZ_AAWY00000000](http://www.ncbi.nlm.nih.gov/genomes/framik.cgi?db=Genome&gi=5616) | 4690501 bp | Proteobacteria; Gammaproteobacteria |  |  |  |
|  | [Shewanella putrefaciens CN-32](http://www.ncbi.nlm.nih.gov/Taxonomy/Browser/wwwtax.cgi?id=319224) |  | [NZ_AALB00000000](http://www.ncbi.nlm.nih.gov/genomes/framik.cgi?db=Genome&gi=5363) | 4530642 bp | Proteobacteria; Gammaproteobacteria |  |  |  |
|  | [Shewanella woodyi ATCC 51908](http://www.ncbi.nlm.nih.gov/Taxonomy/Browser/wwwtax.cgi?id=392500) |  | [NZ_AAUO00000000](http://www.ncbi.nlm.nih.gov/genomes/framik.cgi?db=Genome&gi=5557) | 5815838 bp | Proteobacteria; Gammaproteobacteria |  |  |  |
|  | [Sphingomonas sp. SKA58](http://www.ncbi.nlm.nih.gov/Taxonomy/Browser/wwwtax.cgi?id=314266) |  | [NZ_AAQG00000000](http://www.ncbi.nlm.nih.gov/genomes/framik.cgi?db=Genome&gi=5481) | 3948000 bp | Proteobacteria; Alphaproteobacteria |  |  |  |
|  | [Sphingomonas wittichii RW1](http://www.ncbi.nlm.nih.gov/Taxonomy/Browser/wwwtax.cgi?id=392499) |  | [NZ_AAVK00000000](http://www.ncbi.nlm.nih.gov/genomes/framik.cgi?db=Genome&gi=5580) | 5961785 bp | Proteobacteria; Alphaproteobacteria |  |  | VHML, λ |
|  | [Stappia aggregata IAM 12614](http://www.ncbi.nlm.nih.gov/Taxonomy/Browser/wwwtax.cgi?id=384765) |  | [NZ_AAUW00000000](http://www.ncbi.nlm.nih.gov/genomes/framik.cgi?db=Genome&gi=5566) | 6561391 bp | Proteobacteria; Alphaproteobacteria |  | φJL001 | VP16C, VHML, λ |
|  | [Sulfitobacter sp. EE-36](http://www.ncbi.nlm.nih.gov/Taxonomy/Browser/wwwtax.cgi?id=52598) |  | [NZ_AALV00000000](http://www.ncbi.nlm.nih.gov/genomes/framik.cgi?db=Genome&gi=5393) | 3547243 bp | Proteobacteria; Alphaproteobacteria |  |  | P-SSM2, P-SSM4 |
|  | [Sulfitobacter sp. NAS-14.1](http://www.ncbi.nlm.nih.gov/Taxonomy/Browser/wwwtax.cgi?id=314267) |  | [NZ_AALZ00000000](http://www.ncbi.nlm.nih.gov/genomes/framik.cgi?db=Genome&gi=5396) | 4002069 bp | Proteobacteria; Alphaproteobacteria |  |  |  |
|  | [Synechococcus sp. BL107](http://www.ncbi.nlm.nih.gov/Taxonomy/Browser/wwwtax.cgi?id=313625) |  | [NZ_AATZ00000000](http://www.ncbi.nlm.nih.gov/genomes/framik.cgi?db=Genome&gi=5528) | 2283377 bp | Cyanobacteria |  |  |  |
|  | [Synechococcus sp. RS9916](http://www.ncbi.nlm.nih.gov/Taxonomy/Browser/wwwtax.cgi?id=221359) |  | [NZ_AAUA00000000](http://www.ncbi.nlm.nih.gov/genomes/framik.cgi?db=Genome&gi=5529) | 2664465 bp | Cyanobacteria |  |  |  |
|  | [Synechococcus sp. RS9917](http://www.ncbi.nlm.nih.gov/Taxonomy/Browser/wwwtax.cgi?id=221360) |  | [NZ_AANP00000000](http://www.ncbi.nlm.nih.gov/genomes/framik.cgi?db=Genome&gi=5425) | 2579542 bp | Cyanobacteria |  |  |  |
|  | [Synechococcus sp. WH 5701](http://www.ncbi.nlm.nih.gov/Taxonomy/Browser/wwwtax.cgi?id=69042) |  | [NZ_AANO00000000](http://www.ncbi.nlm.nih.gov/genomes/framik.cgi?db=Genome&gi=5427) | 3043834 bp | Cyanobacteria |  |  |  |
|  | [Synechococcus sp. WH 7805](http://www.ncbi.nlm.nih.gov/Taxonomy/Browser/wwwtax.cgi?id=59931) |  | [NZ_AAOK00000000](http://www.ncbi.nlm.nih.gov/genomes/framik.cgi?db=Genome&gi=5437) | 2620367 bp | Cyanobacteria |  |  |  |
|  | [Tenacibaculum sp. MED152](http://www.ncbi.nlm.nih.gov/Taxonomy/Browser/wwwtax.cgi?id=313598) |  | [NZ_AANA00000000](http://www.ncbi.nlm.nih.gov/genomes/framik.cgi?db=Genome&gi=5416) | 2967100 bp | Bacteroidetes/Chlorobi group |  |  |  |
|  | [Thermoanaerobacter ethanolicus ATCC 33223](http://www.ncbi.nlm.nih.gov/Taxonomy/Browser/wwwtax.cgi?id=340099) |  | [NZ_AAKQ00000000](http://www.ncbi.nlm.nih.gov/genomes/framik.cgi?db=Genome&gi=5352) | 2282740 bp | Firmicutes |  |  |  |
|  | [Thermoanaerobacter ethanolicus X514](http://www.ncbi.nlm.nih.gov/Taxonomy/Browser/wwwtax.cgi?id=399726) |  | [NZ_AATV00000000](http://www.ncbi.nlm.nih.gov/genomes/framik.cgi?db=Genome&gi=5523) | 2275258 bp | Firmicutes |  |  |  |
|  | [Thermosinus carboxydivorans Nor1](http://www.ncbi.nlm.nih.gov/Taxonomy/Browser/wwwtax.cgi?id=401526) |  | [NZ_AAWL00000000](http://www.ncbi.nlm.nih.gov/genomes/framik.cgi?db=Genome&gi=5604) | 2889774 bp | Firmicutes |  |  | φJL001 |
|  | [Thermosipho melanesiensis BI429](http://www.ncbi.nlm.nih.gov/Taxonomy/Browser/wwwtax.cgi?id=391009) |  | [NZ_AAWH00000000](http://www.ncbi.nlm.nih.gov/genomes/framik.cgi?db=Genome&gi=5600) | 1873803 bp | Thermotogae |  |  | S-PM2, KVP40, P-SSM2, P-SSM4 |
|  | [Thermotoga petrophila RKU-1](http://www.ncbi.nlm.nih.gov/Taxonomy/Browser/wwwtax.cgi?id=390874) |  | [NZ_AAWB00000000](http://www.ncbi.nlm.nih.gov/genomes/framik.cgi?db=Genome&gi=5593) | 1788563 bp | Thermotogae |  |  |  |
|  | [Vibrio alginolyticus 12G01](http://www.ncbi.nlm.nih.gov/Taxonomy/Browser/wwwtax.cgi?id=314288) |  | [NZ_AAPS00000000](http://www.ncbi.nlm.nih.gov/genomes/framik.cgi?db=Genome&gi=5470) | 5160431 bp | Proteobacteria; Gammaproteobacteria |  | VHML, λ | VP16C, VHML, λ |
|  | [Vibrio angustum S14](http://www.ncbi.nlm.nih.gov/Taxonomy/Browser/wwwtax.cgi?id=314292) |  | [NZ_AAOJ00000000](http://www.ncbi.nlm.nih.gov/genomes/framik.cgi?db=Genome&gi=5465) | 5101447 bp | Proteobacteria; Gammaproteobacteria |  |  |  |
|  | [Vibrio cholerae 1587](http://www.ncbi.nlm.nih.gov/Taxonomy/Browser/wwwtax.cgi?id=412966) |  | [NZ_AAUR00000000](http://www.ncbi.nlm.nih.gov/genomes/framik.cgi?db=Genome&gi=5562) | 4137501 bp | Proteobacteria; Gammaproteobacteria |  | P2 | P2, S-PM2, P-SSM2, P-SSM4 |
|  | [Vibrio cholerae 2740-80](http://www.ncbi.nlm.nih.gov/Taxonomy/Browser/wwwtax.cgi?id=412614) |  | [NZ_AAUT00000000](http://www.ncbi.nlm.nih.gov/genomes/framik.cgi?db=Genome&gi=5607) | 3945478 bp | Proteobacteria; Gammaproteobacteria |  |  |  |
|  | [Vibrio cholerae B33](http://www.ncbi.nlm.nih.gov/Taxonomy/Browser/wwwtax.cgi?id=417400) |  | [NZ_AAWE00000000](http://www.ncbi.nlm.nih.gov/genomes/framik.cgi?db=Genome&gi=5597) | 4026835 bp | Proteobacteria; Gammaproteobacteria |  | P2 | P2 |
|  | [Vibrio cholerae MAK 757](http://www.ncbi.nlm.nih.gov/Taxonomy/Browser/wwwtax.cgi?id=412967) |  | [NZ_AAUS00000000](http://www.ncbi.nlm.nih.gov/genomes/framik.cgi?db=Genome&gi=5563) | 3917446 bp | Proteobacteria; Gammaproteobacteria |  |  |  |
|  | [Vibrio cholerae MO10](http://www.ncbi.nlm.nih.gov/Taxonomy/Browser/wwwtax.cgi?id=345072) |  | [NZ_AAKF00000000](http://www.ncbi.nlm.nih.gov/genomes/framik.cgi?db=Genome&gi=5348) | 4024286 bp | Proteobacteria; Gammaproteobacteria |  | P2 | P2 |
|  | [Vibrio cholerae MZO-3](http://www.ncbi.nlm.nih.gov/Taxonomy/Browser/wwwtax.cgi?id=412883) |  | [NZ_AAUU00000000](http://www.ncbi.nlm.nih.gov/genomes/framik.cgi?db=Genome&gi=5564) | 4146039 bp | Proteobacteria; Gammaproteobacteria |  |  | S-PM2, P-SSM2, P-SSM4 |
|  | [Vibrio cholerae NCTC 8457](http://www.ncbi.nlm.nih.gov/Taxonomy/Browser/wwwtax.cgi?id=417399) |  | [NZ_AAWD00000000](http://www.ncbi.nlm.nih.gov/genomes/framik.cgi?db=Genome&gi=5596) | 4063388 bp | Proteobacteria; Gammaproteobacteria |  | P2 | P2, S-PM2, P-SSM2, P-SSM4 |
|  | [Vibrio cholerae O395](http://www.ncbi.nlm.nih.gov/Taxonomy/Browser/wwwtax.cgi?id=345073) |  | [NZ_AAKG00000000](http://www.ncbi.nlm.nih.gov/genomes/framik.cgi?db=Genome&gi=5347) | 4148680 bp | Proteobacteria; Gammaproteobacteria |  | P2 | P2 |
|  | [Vibrio cholerae RC385](http://www.ncbi.nlm.nih.gov/Taxonomy/Browser/wwwtax.cgi?id=345074) |  | [NZ_AAKH00000000](http://www.ncbi.nlm.nih.gov/genomes/framik.cgi?db=Genome&gi=5346) | 3625004 bp | Proteobacteria; Gammaproteobacteria |  |  | S-PM2, P-SSM2, P-SSM4 |
|  | [Vibrio cholerae V51](http://www.ncbi.nlm.nih.gov/Taxonomy/Browser/wwwtax.cgi?id=345075) |  | [NZ_AAKI00000000](http://www.ncbi.nlm.nih.gov/genomes/framik.cgi?db=Genome&gi=5345) | 3773421 bp | Proteobacteria; Gammaproteobacteria |  |  |  |
|  | [Vibrio cholerae V52](http://www.ncbi.nlm.nih.gov/Taxonomy/Browser/wwwtax.cgi?id=345076) |  | [NZ_AAKJ00000000](http://www.ncbi.nlm.nih.gov/genomes/framik.cgi?db=Genome&gi=5344) | 3906793 bp | Proteobacteria; Gammaproteobacteria |  |  |  |
|  | [Vibrio sp. Ex25](http://www.ncbi.nlm.nih.gov/Taxonomy/Browser/wwwtax.cgi?id=150340) |  | [NZ_AAKK00000000](http://www.ncbi.nlm.nih.gov/genomes/framik.cgi?db=Genome&gi=5349) | 4839877 bp | Proteobacteria; Gammaproteobacteria |  |  |  |
|  | [**Vibrio sp. MED222**](http://www.ncbi.nlm.nih.gov/Taxonomy/Browser/wwwtax.cgi?id=314290) |  | [**NZ_AAND00000000**](http://www.ncbi.nlm.nih.gov/genomes/framik.cgi?db=Genome&gi=5419) | **4891901 bp** | **Proteobacteria; Gammaproteobacteria** | **7379-20020 (AAND01000006)** |  |  |
|  | [**Vibrio splendidus 12B01**](http://www.ncbi.nlm.nih.gov/Taxonomy/Browser/wwwtax.cgi?id=314291) |  | [**NZ_AAMR00000000**](http://www.ncbi.nlm.nih.gov/genomes/framik.cgi?db=Genome&gi=5401) | **5596386 bp** | **Proteobacteria; Gammaproteobacteria** | **7583-19683 (AAMR01000034)** | P2 | P2, VHML, λ |
|  | [**Vibrionales bacterium SWAT-3**](http://www.ncbi.nlm.nih.gov/Taxonomy/Browser/wwwtax.cgi?id=391574) |  | [**AAZW00000000**](http://www.ncbi.nlm.nih.gov/entrez/viewer.fcgi?db=nucleotide&val=145965950) | **5830607 bp** | **Proteobacteria; Gammaproteobacteria** | **9390-22325**  **(AAZW01000031)** |  |  |
|  | [Xanthobacter autotrophicus Py2](http://www.ncbi.nlm.nih.gov/Taxonomy/Browser/wwwtax.cgi?id=78245) |  | [NZ_AAPC00000000](http://www.ncbi.nlm.nih.gov/genomes/framik.cgi?db=Genome&gi=5458) | 5580887 bp | Proteobacteria; Alphaproteobacteria |  |  | P2, P-SSM4 |
|  | [Yersinia frederiksenii ATCC 33641](http://www.ncbi.nlm.nih.gov/Taxonomy/Browser/wwwtax.cgi?id=349966) |  | [NZ_AALE00000000](http://www.ncbi.nlm.nih.gov/genomes/framik.cgi?db=Genome&gi=5367) | 4845578 bp | Proteobacteria; Gammaproteobacteria |  | P2 | P2, P-SSM2 |
|  | [Yersinia intermedia ATCC 29909](http://www.ncbi.nlm.nih.gov/Taxonomy/Browser/wwwtax.cgi?id=349965) |  | [NZ_AALF00000000](http://www.ncbi.nlm.nih.gov/genomes/framik.cgi?db=Genome&gi=5368) | 4677210 bp | Proteobacteria; Gammaproteobacteria |  |  | VP16C, VHML, λ |
|  | [Yersinia mollaretii ATCC 43969](http://www.ncbi.nlm.nih.gov/Taxonomy/Browser/wwwtax.cgi?id=349967) |  | [NZ_AALD00000000](http://www.ncbi.nlm.nih.gov/genomes/framik.cgi?db=Genome&gi=5366) | 4527725 bp | Proteobacteria; Gammaproteobacteria |  |  |  |

^a^ - Organisms in which chromosomes corticoviral elements were identified are shown in **bold**

^b^ - Subphyla are listed for proteobacteria

^c^ - Shotgun library segment is indicated in the parenthesis
